# The golden genome annotation of *Ganoderma lingzhi* reveals a more complex scenario of eukaryotic gene structure and transcription activity

**DOI:** 10.1186/s12915-024-02073-y

**Published:** 2024-11-25

**Authors:** Lining Wang, Peiqi Shi, Zhaohua Ping, Qinghua Huang, Liqun Jiang, Nianfang Ma, Qingfu Wang, Jiang Xu, Yajie Zou, Zhihai Huang

**Affiliations:** 1 Guangdong Engineering Laboratory of Biomass Value-added Utilization, Guangdong Engineering Research & Development Center for Comprehensive Utilization of Plant Fiber, Guangzhou Key Laboratory for Comprehensive Utilization of Plant Fiber, Institute of Biological and Medical Engineering, Guangdong Academy of Sciences, Guangzhou, 510316 China; 2 The Second Clinical College, Guangzhou University of Chinese Medicine, Guangzhou, 510120 China; 3 Institute of Chinese Materia Medica, China Academy of Chinese Medical Sciences, Beijing, 100700 China; 4 Institute of Agricultural Resources and Regional Planning, Chinese Academy of Agricultural Sciences, Beijing, 100081 China

**Keywords:** *G. lingzhi*, Non-canonical splicing sites, Overlapped genes, Alternative splicing, Eukaryotic polycistronic genes

## Abstract

**Background:**

It is generally accepted that nuclear genes in eukaryotes are located independently on chromosomes and expressed in a monocistronic manner. However, accumulating evidence suggests a more complex landscape of gene structure and transcription. *Ganoderma lingzhi*, a model medicinal fungus, currently lacks high-quality genome annotation, hindering genetic studies.

**Results:**

Here, we reported a golden annotation of *G. lingzhi*, featuring 14,147 high-confidence genes derived from extensive manual corrections. Novel characteristics of gene structure and transcription were identified accordingly. Notably, non-canonical splicing sites accounted for 1.99% of the whole genome, with the predominant types being GC-AG (1.85%), GT-AC (0.05%), and GT-GG (0.04%). 1165 pairs of genes were found to have overlapped transcribed regions, and 92.19% of which showed opposite directions of gene transcription. A total of 5,412,158 genetic variations were identified among 13 *G. lingzhi* strains, and the manually corrected gene sets resulted in enhanced functional annotation of these variations. More than 60% of *G. lingzhi* genes were alternatively spliced. In addition, we found that two or more protein-coding genes (PCGs) can be transcribed into a single RNA molecule, referred to as polycistronic genes. In total, 1272 polycistronic genes associated with 2815 PCGs were identified.

**Conclusions:**

The widespread presence of polycistronic genes in *G. lingzhi* strongly complements the theory that polycistron is also present in eukaryotic genomes. The extraordinary gene structure and transcriptional activity uncovered through this golden annotation provide implications for the study of genes, genomes, and related studies in *G. lingzhi* and other eukaryotes.

**Supplementary Information:**

The online version contains supplementary material available at 10.1186/s12915-024-02073-y.

## Background

*Ganoderma lingzhi*, long considered synonymous with *G. lucidum* [1], is one of the most well-known medicinal macro-fungi in the world. It has consistently attracted research interest and serves as a model organism for studying medicinal fungi, with ongoing reports detailing its pharmacological activities. From the first genome map published in 2012 [2], more and more genomes of *Ganoderma* are available now [3–5]. Although relatively high-quality assemblies are now accessible, the quality of genome annotation can be significantly influenced by various factor, including software used, rounds of reannotation, reference protein databases, and RNA sequencing (RNA-Seq) data. Accurate and complete gene annotation is an important aspect of a reliable reference genome, yet it is often overlooked. In our previous study, we found that manual corrections to gene structures, based on RNA-Seq data, enhance the accuracy of subsequent bioinformatics analyses and gene cloning efforts [6].

To achieve more accurate and complete annotations, genomes typically undergo several rounds of reannotation. Notable examples include the community-driven updates of the *Aspergillus nidulans* [7], the release of 11th annotation of the *Arabidopsis thaliana* genome in 2017 [8], and the updated annotation of the wild strawberry *Fragaria vesca* V4 genome in 2019 [9]. These updates mainly depend on abundant RNA-Seq data, new annotation methods, or combined analytical methods, resulting in the identification of numerous new genes. These escalating annotated versions of the genome have greatly facilitated scientific research and enhanced our understanding of genomic landscapes. Reannotation using software relies heavily on existing gene models, yet a significant portion of the gene pool remains understudied, raising the possibility that many already annotated genes may contain errors. Besides, gene structures may vary greatly between species and even among different varieties, which can lead to inaccurate annotation results. Therefore, manual correction using transcriptome data has emerged as a relatively precise and efficient method for structurally correcting a high number of genes.

A key feature of eukaryotic gene structure is the presence of exons and introns within their transcripts. Exon regions are retained in the final mature mRNA molecule, while intron regions are spliced out during post-transcriptional processing which involves small nuclear RNAs and spliceosomes [10]. The dinucleotides, GT-AG, are called canonical splicing sites or canonical introns, and those differing from GT-AG are called non-canonical splicing sites. Numerous non-canonical splicing sites have been identified, including GC-AG, AT-AC, GT-TG, GG-AG, and AT-AG, with GC-AG being the most prevalent non-canonical splicing sites, followed by AT-AC [11, 12]. While the landscape of non-canonical splicing sites in plants and humans has been well studied [11, 12], macro-fungi have received relatively little attention in this regard.

Alternative splicing (AS), the differential processing of introns and exons in pre-mRNAs to produce multiple transcript isoforms per gene, is the most important contributor to transcriptome diversification in eukaryotes [13]. AS can enhance proteome complexity by generating two or more distinct protein isoforms and can effectively lead to downregulation of gene expression by creating truncated protein isoforms [14]. According to RNA-Seq analyses, the ratio of multiexonic genes undergoing AS exceeds 95% in humans [15], 42.4% in rice [16], and 61% in *Arabidopsis* [17]. However, fungi generally exhibit a much lower AS ratio, with an average of only 6.4% of annotated genes affected by AS across a study of 23 fungal species [18], a significant increase from earlier reports of 1.6–3.6% [19]. Nonetheless, deep RNA-Seq reveals that 48.9% of genes are alternatively spliced in *Trichoderma longibrachiatum* [20], indicating an underestimation of AS in fungi. Compared to higher organisms, our understanding of AS in fungi is still limited, particularly concerning its prevalence, molecular functions, and regulatory mechanisms in mushroom-forming fungi.

It is generally believed that eukaryotic genes are positionally isolated in genome and transcribed into different RNA molecules. However, recent studies have revealed a much more complex scenario of gene structures and transcription activities. Eukaryotic genes can have overlapping regions with adjacent genes [21], and in some cases, multiple genes are transcribed into a single RNA molecule [22]. Similar to prokaryotic operons, two or more genes can be transcribed as a single polycistronic mRNA or can also been transcribed independently in eukaryotes, and these polycistronic transcripts are called polycistrons [23]. In 2015, Gordon et al. reported for the first time that polycistronic transcription is prevalent in the genome of higher fungi and especially prevalent among mushroom-forming Agaricomycetes, such as *Trametes versicolor* and *Gloeophyllum trabeum* [24]. Nevertheless, systematic studies of polycistronic genes in macro-fungi remain scarce.

Here, we report two haplotype-genomes of *G. lingzhi* strain GL0102, designated GL0102_8 and GL0102_53, respectively. And we get a high-quality annotation by manually correcting all genes with the help of full-length transcriptome and RNA-Seq in GL0102_53. Our annotation revealed a considerable number of non-canonical splicing sites, overlapped genes, AS, and polycistronic genes. The updated annotation, novel features of gene structure, and gene transcription provide useful resources for research in other macro-fungi and deepen our understanding of how genetic information is encoded in the genome of macro-fungi.

## Results

### Genome assembly, annotation and curation of *G. lingzhi*

**Fig. 1:**
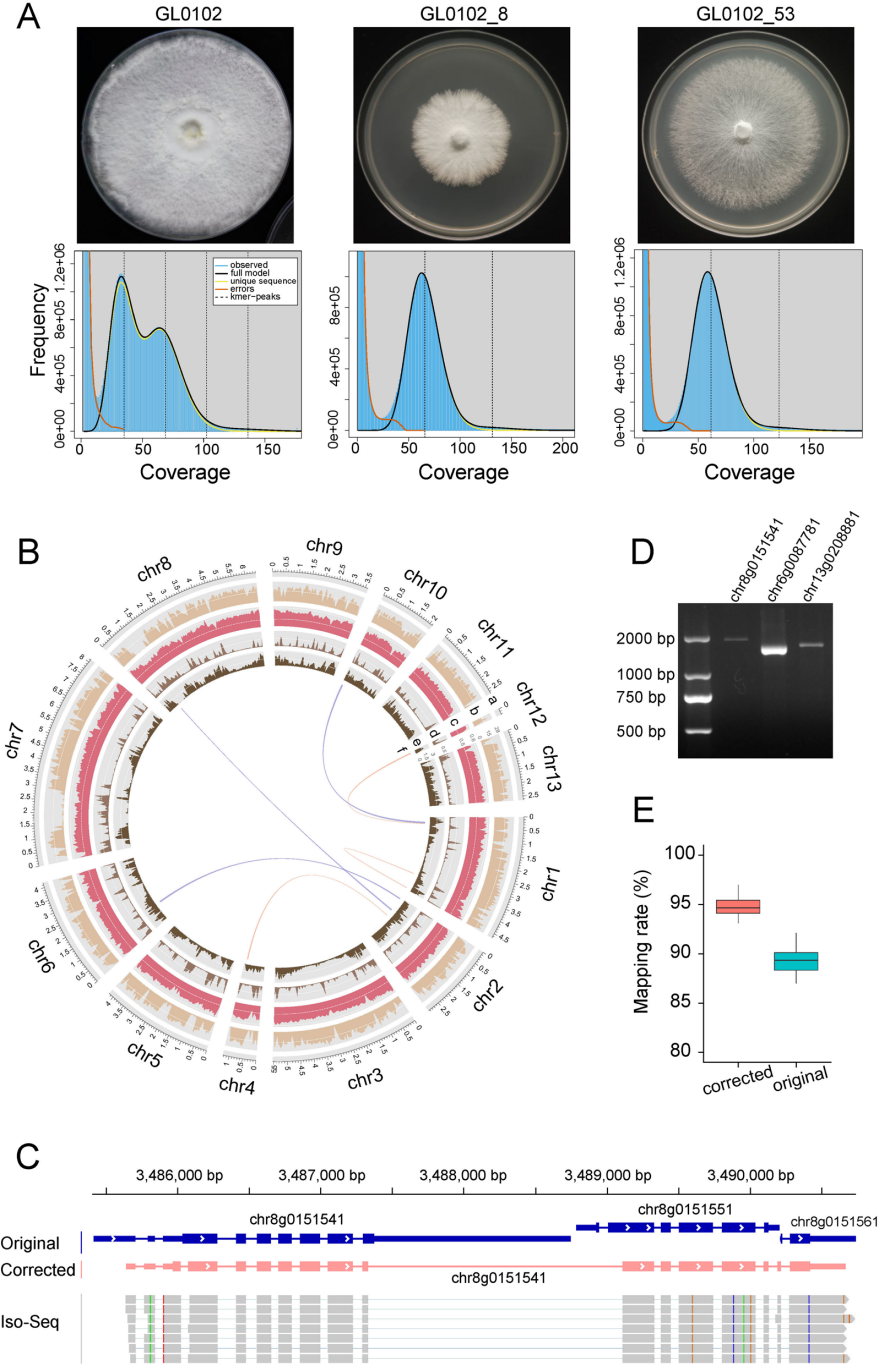
Genome survey, genome assembly, and gene correction. **A** K-mer analysis of genome survey and mycelia of *G. lingzhi*. **B** Global view of the GL0102_53 genome. a, chromosomes; b, gene density; c, GC contents; d, repeat contents; e, variant density in GL0102_53 versus GL0102_8; f, syntenic blocks (sequence length, ≥ 5 kb). The average GC content of the GL0102_53 genome was 55.72%. Notably, regions with a higher proportion of repeat sequences displayed fewer genes and lower GC content. All statistics were calculated over 50 kb non-overlapping windows. **C** Gene structure of a P450 gene before and after correction. Arrows indicate the direction of transcription. **D** Electrophoresis of amplification products of P450 genes. **E** RNA-Seq read mapping rates against the mRNA sequences of GL0102_53, both before and after correction

Whole genome sequencing on Illumina and PacBio Sequel platforms was performed on both dikaryon (GL0102) and monokaryon (GL0102_8 and GL0102_53) strains (Additional file 1: Table S1). The genome size was estimated to be 43.96–46.74 Mb in all strains, and the heterozygosity was 1.55% in genome of dikaryon strain (Fig. 1A). GL0102_8 and GL0102_53 were assembled into 13 chromosomes, with total lengths of 46.35 Mb (N50 4.97 Mb) and 48.56 Mb (N50 4.67 Mb), respectively (Fig. 1B, Additional file 1: Table S2). GL0102_8 and GL0102_53 exhibited 13.46% and 17.97% repeat sequences, respectively (Additional file 1: Table S3). No fragment loss was revealed by KAT (Additional file 1: Fig. S1), and a high concordance of over 99.4% was revealed by proovframe (Additional file 1: Table S2). The Benchmarking Universal Single-Copy Ortholog (BUSCO) completeness was 99.1% (Additional file 1: Table S4). RNA-Seq, isoform sequencing (Iso-Seq), and proteomics were performed on mycelia (M), early primordia (Pe), and late primordia (Pl) (Additional file 1: Table S5), and these data were used in subsequent gene prediction and correction. The gene annotation of GL0102_8 and GL0102_53 was conducted using EuGene [25], and then all genes of GL0102_53 genome were manually corrected using Apollo [26]. According to the support of RNA-Seq and Iso-Seq transcripts, the gene structures were manually corrected. As a result, 4277 full-match genes remained unchanged, 26,975 exons were deleted, 6175 novel exons were added, 23,681 introns were deleted, 9718 novel introns were added, 6369 loci were deleted, and 990 novel loci were added. Finally, 14,147 high-confidence genes were annotated in the manually corrected genome of GL0102_53.

During the manual correction of the GL0102_53 genome, we found that there were a lot mistakes in tool-predicted gene structures, even for the well-studied gene families. For example, a P450 gene was reconstructed by combining three separate genes (chr8g0151541, Fig. 1C). The 3′ region of a P450 gene was rescued by adding four introns and four exons (chr6g0087771, Additional file 1: Fig. S2A), and the 5′ region of a P450 gene was rescued by adding two introns and two exons (chr13g0208881, Additional file 1: Fig. S2B). Moreover, the manually corrected P450 gene structures were confirmed by PCR amplification and Sanger sequencing (Fig. 1D). All the genes in the manually corrected genome of GL0102_53 were searched against the UniProt database, and 393 genes were found to have no hit at all, of which only 15 had PFAM domain annotations. Among these 393 genes, 83 were novel genes discovered during the manual correction process.

The original and corrected gene sets were assessed at various levels. The completeness assessed by BUSCO analysis increased from 89.7 to 99.1% after correction (Additional file 1: Table S4). The proportion of genes that can be annotated was significantly increased in the corrected gene sets. Specifically, approximately 72.63% of genes in the corrected gene sets were annotated by EggNOG, 52.7% by Pfam, and 49.91% by UniProt (Additional file 1: Table S6). In addition, RNA-Seq data of M, Pe, and Pl were mapped to the mRNA sequences of both the original and corrected gene sets. Compared to the original gene set, the mapping rate of the corrected gene sets increased by nearly 5% (Fig. 1E), indicating a more accurate calculation of gene expression levels when using the corrected gene sets as a reference.

### Rich non-canonical splicing sites were identified in the genome of *G. lingzhi*

Splicing sites, which consisted of a pair of donor and acceptor nucleotides, define the boundaries of exon and intron and can be classified into two categories: canonical and non-canonical. In the original gene sets, canonical splicing sites accounted for 98.01%, while non-canonical splicing sites made up 1.99% (GC-AG 1.26%, GT-AC 0.72%, and GC-AC 0.01%). In corrected gene sets, however, more types and higher rate of non-canonical splicing sites were discovered. Specifically, 97.56% of the splicing sites were GT-AG, and the rest were 94 non-canonical splice sites (2.44%) representing 16 types of donors and 16 types of acceptors (Table 1 and Additional file 1: Table S7). Among all the non-canonical splicing sites, 20 types showed a proportion greater than 0.01%, with GC-AG (1.85%), GT-AC (0.05%), and GT-GG (0.04%) being the three most abundant non-canonical splicing sites (Table 1). Non-canonical splicing sites were distributed in all the 13 chromosomes and were involved in 1847 genes. And genes containing non-canonical splicing sites have no significant functional enrichment. A single pair of non-canonical splicing sites was identified in 1653 genes, while two or more pairs were identified in 194 genes. For instance, chr5g0072011 (SNF2 family N-terminal domain-containing protein, Additional file 1: Fig. S3A) contained three GC-AG splicing sites, whereas chr5g0071881 (uncharacterized protein, Additional file 1: Fig. S3B) had two.

**Table 1:** Diversity of *G. lingzhi* splicing sites

Type	Number	Ratio	Type	Number	Ratio
GT-AG	89,855	97.555000	GG-AG	20	0.021714
GC-AG	1706	1.852190	GT-CG	19	0.020628
GT-AC	49	0.053199	GT-AT	18	0.019542
GT-GG	38	0.041256	CT-AG	17	0.018457
GA-AG	35	0.037999	GT-GT	17	0.018457
GT-TG	22	0.023885	GT-CT	15	0.016285
GT-AA	22	0.023885	AT-AC	13	0.014114
GT-GC	21	0.022800	GT-TC	11	0.011943
GT-CC	21	0.022800	GT-TA	10	0.010857
CA-AG	21	0.022800	Others	156	0.169368
AT-AG	21	0.022800			

**Fig. 2:**
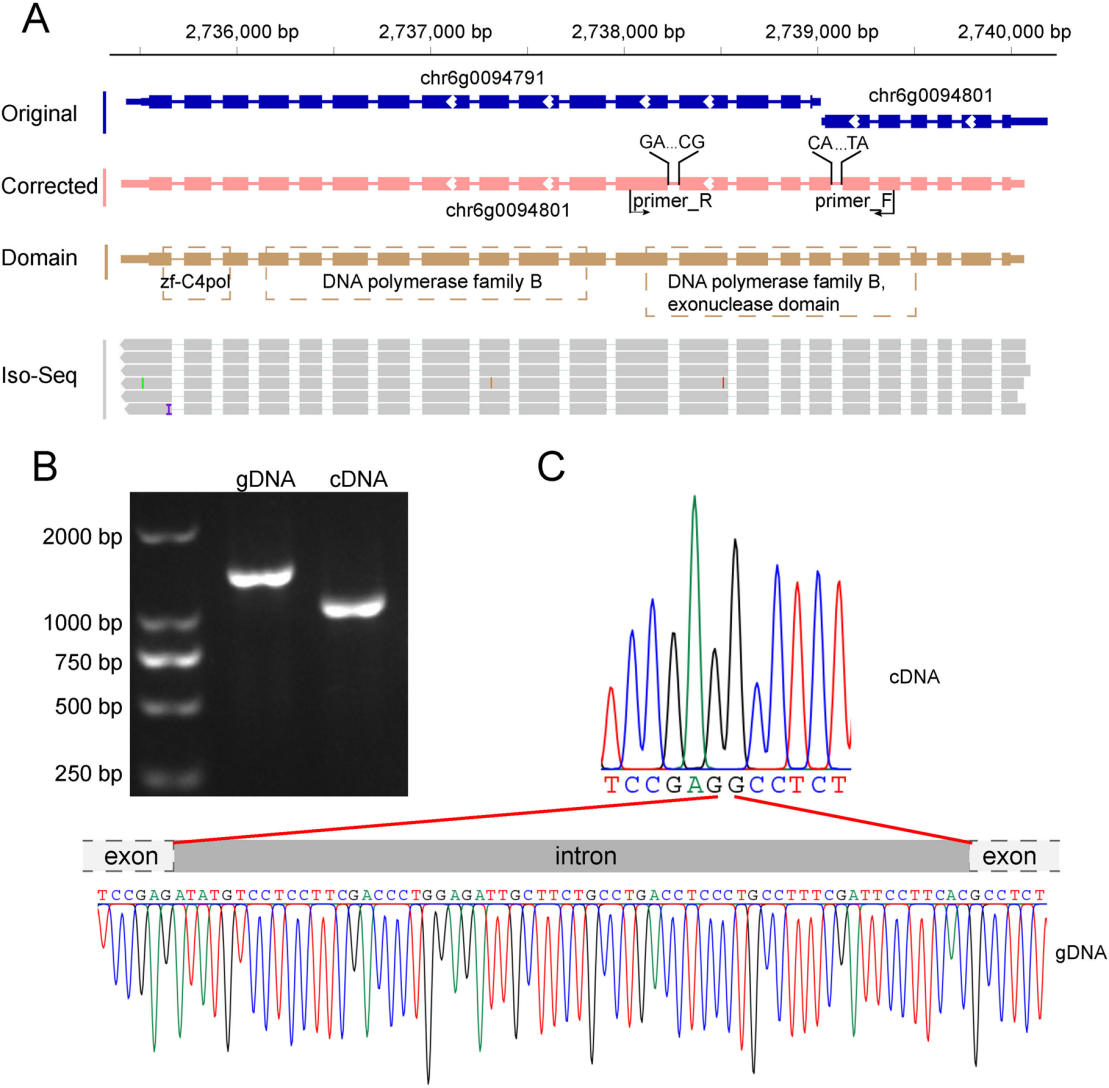
Non-canonical splicing sites in *G. lingzhi.* **A** AT-AC and GC-AG splicing sites in chr6g0094801. Arrows indicate the direction of transcription. zf-C4pol, C4-type zinc-finger of DNA polymerase delta. **B** Agarose gel electrophoresis of PCR products featuring AT-AC and GC-AG splicing sites. **C** Sanger sequencing of PCR products containing AT-AC splicing sites

The inadequate recognition of non-canonical splicing signals may negatively impact gene structural predictions and functional annotations. For example, in the original gene sets, the interrupted chr6g0094801 resulted in incomplete functional domains due to a failure to recognize the AT-AC splicing sites (Fig. 2A). A pair of primers was designed to amplify the flanking regions of the AT-AC splicing sites of chr6g0094801 (Fig. 2B and Additional file 1: Table S8). The AT-AC as well as an additional GC-AG splicing sites were confirmed by Sanger sequencing (Fig. 2C). In addition, 10 types of non-canonical splicing sites were randomly selected and confirmed (Additional file 1: Table S8 and Additional file 1: Fig. S3C and 3D).

### Compelling overlapped genes existed in the genome of *G. lingzhi*

**Fig. 3:**
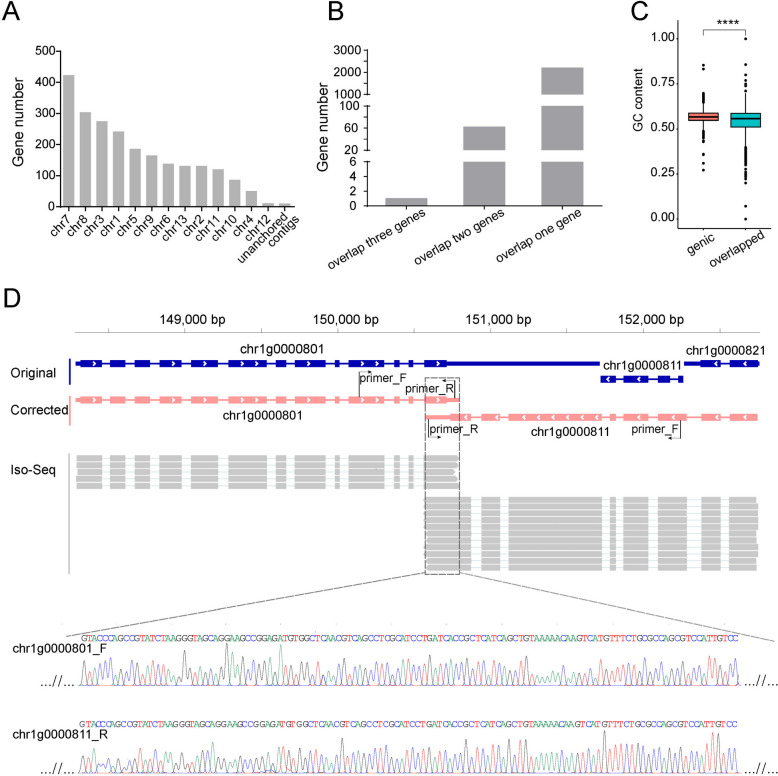
Statistics and validation of overlapped genes. **A** Distribution of overlapped genes across different chromosomes (chr). **B** Classification of overlapped genes. **C** GC contents of overlapped regions and genic regions of the genome. “****” significant difference, *T-*test, *P* < 2.2e − 16. **D** Confirmation of overlapped region. Arrows indicate the direction of transcription. The dashed rectangular box indicates the overlapped region

The present gene prediction tools were designed to produce genes with no overlapping in eukaryotic genomes, while emerging evidence revealed the existence of overlapped genes in eukaryotic organisms [21, 27]. In fact, no overlapped gene was predicted in *G. lingzhi* genome by gene prediction tools used in this study or previous studies [2–5]. However, during manual correction, abundant overlapped genes supported by full length transcripts were discovered in *G. lingzhi*. In the genome of GL0102_53, 1165 pairs of genes were found to have overlapped transcribed regions, involving a total of 2266 genes located across all 13 chromosomes (Fig. 3A). Most of these genes overlapped one by one, while 63 genes overlapped with two or three genes (Fig. 3B). Among the overlapped gene pairs, 1074 pairs showed opposite gene transcription directions. The majority of these overlapped genes showed overlap at the 3′-terminus (73.47%), with the average proportion of overlapped regions relative to the full length of the genes being 26.70%. Additionally, there were 146 genes located within other genes but oriented in opposite directions. Overlapped regions had significant lower GC content (54.20%) than that of all genic regions of the genome (56.82%) (Fig. 3C). The overlapped genes showed no significant functional enrichment. The overlapped region between a pair of overlapped genes, chr1g0000801 (p450) and chr1g0000811(SET domain-containing protein), was confirmed by PCR amplification and Sanger sequencing using cDNA of GL0102 as the amplification template (Fig. 3D).

### Prevalent genetic variations presented among *G. lingzhi* strains

**Fig. 4:**
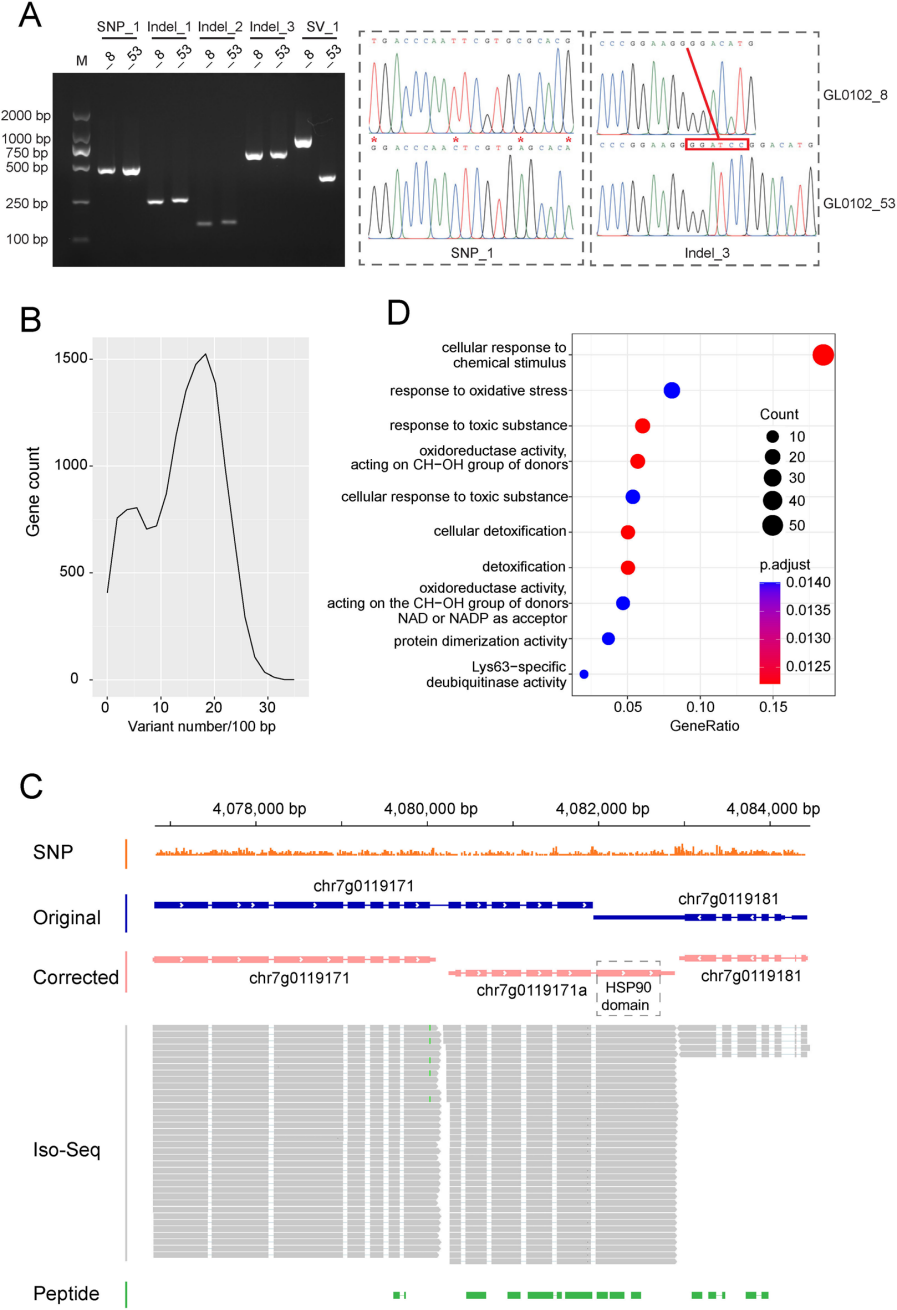
Genetic variations in *G. lingzhi.* **A** Confirmation of genetic variations between GL0102_8 and GL0102_53. “_8” and “_53” represent GL0102_8 and GL0102_53, respectively. Red stars represent SNP loci. Red rectangular box represents inserted sequences in GL0102_53. **B** Density of variations in genic region. **C** SNPs in a *HSP90* gene (chr7g0119171a). Arrows indicate the direction of transcription. SNPs were displayed in 10 bp non-overlapping windows. **D** Functional enrichment of conserved genes. Count represents the number of genes annotated under the same Gene Ontology (GO) term, p.adjust represents statistical testing metrics, and GeneRatio represents ratio of input genes that are annotated in a term

Strains or haploids of *G. lingzhi* varied in phenotypes. For example, when cultured on PDA plates, GL0102_53 demonstrated a faster growth rate and sparser mycelia, while GL0102_8 showed a slower growth rate and denser mycelia (Fig. 1A). The availability of high-quality genomes makes phenotype-genotype relationship investigation feasible by comparative genomics. In this study, 12 *G. lingzhi* genomes were compared to the reference genome GL0102_53. A total of 5,412,158 genetic variations were identified, with single-nucleotide polymorphisms (SNPs) being the most prevalent, numbering 4,919,524 (90.90%). Additionally, there were 483,420 insertion/deletions (indels) and 9214 structural variations (SVs). Among all variations, 418,180 SNPs, 53,160 indels, and 2257 SVs were discovered between GL0102_53 and GL0102_8, with 59 SNPs, 23 indels, and 7 SVs confirmed by PCR amplification and Sanger sequencing (Fig. 4A and Additional file 1: Table S9). Of the identified genetic variations, 722,123 located or overlapped with non-genic regions (density of 4.42/100 bp), while 4,694,470 were found in or overlapping with genic regions (density of 14.09/100 bp, Fig. 4B). Furthermore, 2,399,162 genetic variations located or overlapped with coding regions (density of 11.55/100 bp). Indels and SVs, which caused significant base changes, occurred less frequently compared to SNPs in coding regions. Overall, 47.34% of SNPs, 14.30% of indels, and 13.60% of SVs in the whole genome were located or overlapped with coding regions. A considerable number (174,868) of variants were found to be located or overlapped with coding regions while they were located in non-coding regions in the original gene annotations, which indicated that the corrected gene sets had an improvement in variant functional annotation. For example, the last exon of chr7g0119171a, which contains an HSP90 domain and exhibits a high density of SNPs among *G. lingzhi* strains, was originally annotated as a UTR of a gene on the opposite strand (Fig. 4C). Conserved genes (density of variation < 10/100 bp) showed functional enrichment in response to stress (Fig. 4D), suggesting that *G. lingzhi* is relatively conservative in its survival strategies. In contrast, no significant functional enrichment was found for highly variable genes (density of variation > 20/100 bp), indicating their potential dispensable roles in survival and their relationship to strain diversity.

### More than half of genes were alternative spliced in *G. lingzhi* genome

**Fig. 5:**
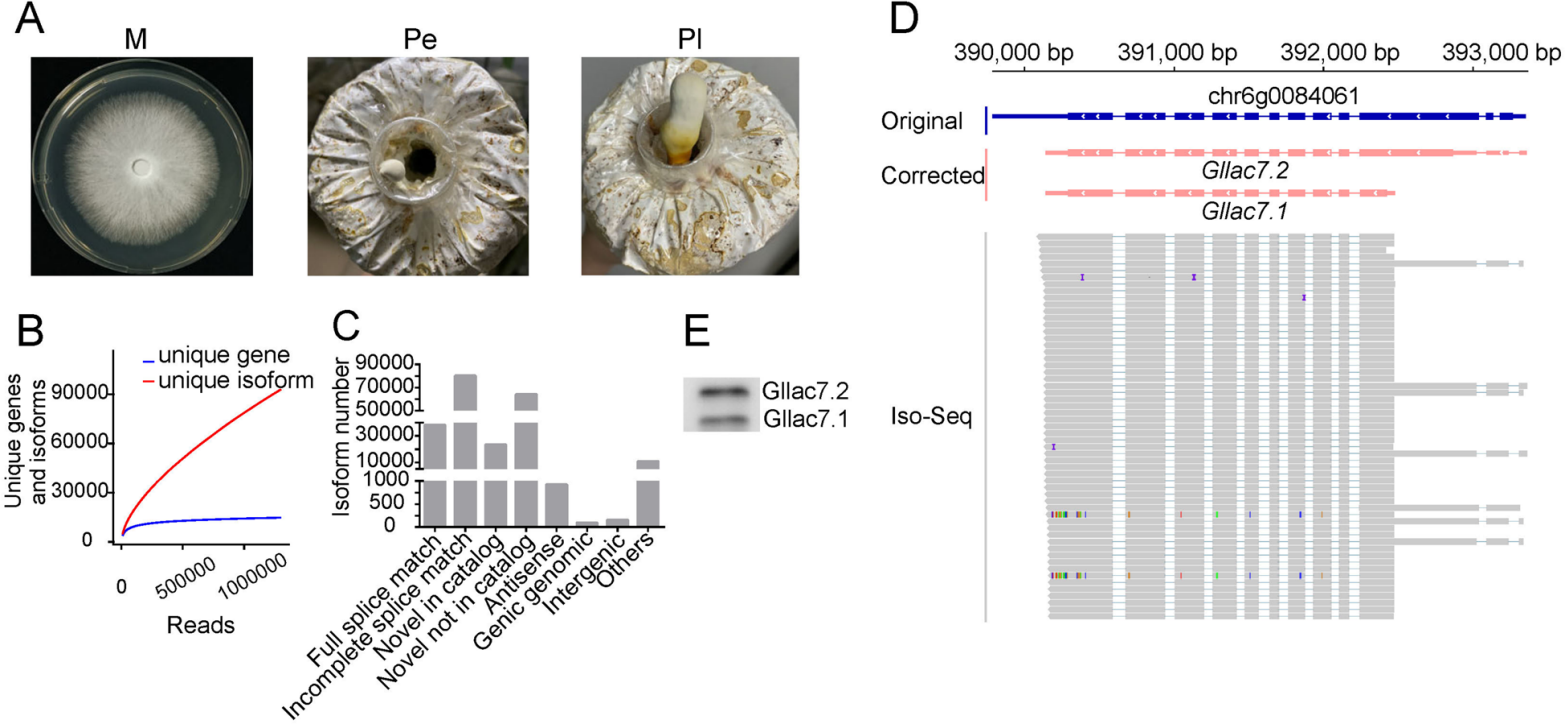
Characteristics of AS in *G. lingzhi.* **A** Samples of different developmental stages of GL0102. M, mycelia; Pe, early primordia; Pl, late primordia. **B** Saturation test for unique gene and isoform detection; **C** Statistic of AS in the GL0102_53 genome; **D** AS in *Gllac7*. Arrows indicate the direction of transcription. **E** Western blot of Gllac7

AS expands transcriptome and proteome diversification/complexity greatly, while the accurate identification of AS is challenging. Facilitated by high-quality annotated genomes and intact transcripts, AS could be captured comprehensively and precisely. To identify AS in *G. lingzhi*, corrected gene annotation and full-length transcripts of M, Pe, and Pl were applied (Fig. 5A). In total, 2.36 Gb, 1.88 Gb, and 1.97 Gb Iso-Seq reads were obtained for M, Pe, and Pl, respectively (Additional file 1: Table S5). And 1.37 million full length transcripts were generated from Iso-Seq reads. As more full-length transcripts used in analysis, more unique genes and unique isoforms could be discovered. The number of unique genes showed little increase once the number of full-length transcripts exceeded 500,000 (Fig. 5B). In contrast, the number of unique isoforms had not reached saturation even with the use of one million full-length transcripts (Fig. 5B), suggesting that many more isoforms remain to be discovered.

A total of 217,321 isoforms belonging to 15,266 unique genes were identified and clustered in eight categories. Among these categories, incomplete splice match was the most with a percent of 36.86%, followed by Novel not in catalog (29.41%), Full splice match (17.53%), Novel in catalog (10.69%), Antisense (0.42%), Intergenic (0.07%), Genic genomic (0.04%), and others (4.97%) (Fig. 5C). In the genome of GL0102_53, 5418 genes showed no AS, while 9848 genes had two or more splice isoforms, and 5470 genes had more than ten splice isoforms. The average AS rate of the whole genome gene set was 14.24 per gene.

In some challenging cases of AS detection, full-length transcript data showed superior performance. For instance, *Gllac7*, a laccase of *G. lingzhi*, had no AS events detected or annotated in the original gene prediction [28], while two isoforms (*Gllac7.1* and *Gllac7.2*) with altered 5′-ends were identified based on full-length transcripts (Fig. 5D). Since *Gllac7.1* is entirely contained within *Gllac7.2*, the two isoforms had identical sequences in their overlapped regions, making them difficult to distinguish with short read sequencing. Beyond the differences in transcript length, the two isoforms also encode peptides of different sizes, as supported by western blot (Fig. 5E). In addition, AS of ten genes were confirmed by PCR amplification and Sanger sequencing (Additional file 1: Table S10 and Additional file 1: Fig. S4).

### Iso-Seq transcripts revealed widespread polycistronic genes in *G. lingzhi*

**Fig. 6:**
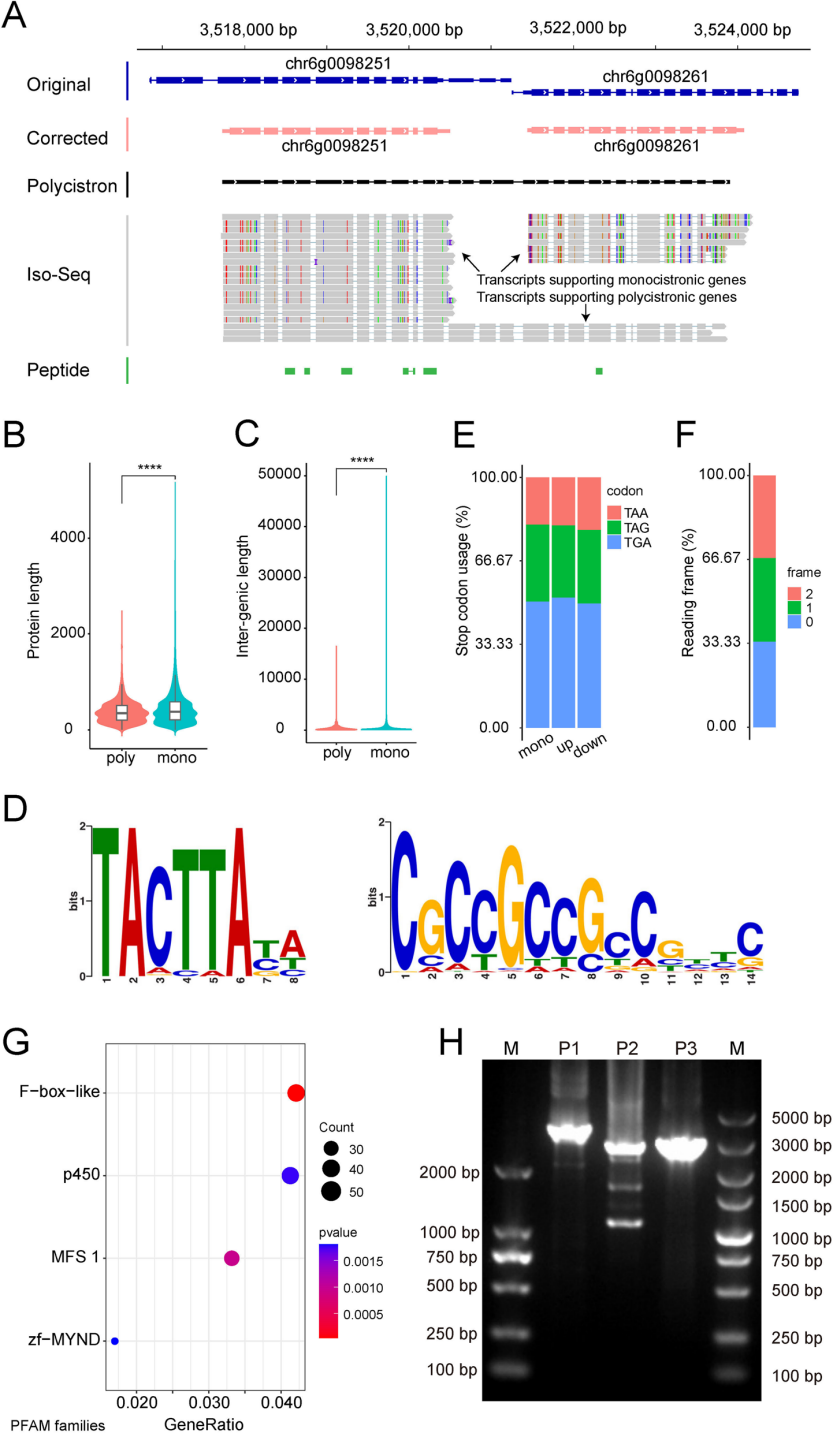
Characteristics of polycistronic genes of *G. lingzhi.* **A** View of a polycistronic gene supported by Iso-Seq transcripts. The Iso-Seq transcripts supports the existence of both polycistronic and monocistronic genes. Peptide evidence and Iso-Seq transcripts indicate that upstream and downstream genes are transcribed and translated separately. **B**, **C** Comparative analysis of protein length and inter-genic length between polycistronic and monocistronic genes. **D** Motifs in the linker regions of the polycistronic genes. **E**, **F** Frequency of stop codon usage and reading frames of polycistronic genes versus monocistronic genes. **G** Functional enrichment of PCGs within polycistronic genes. Count represents the number of genes annotated under the same PFAM family, pvalue represents statistical testing metrics, and GeneRatio represents ratio of input genes that are annotated in a PFAM family. **H** Agarose gel electrophoresis of PCR products of polycistronic genes. The predicted sizes of the targeted PCR products were 3610, 2675, and 3019 bp, respectively. “poly” and “mono” represent “polycistronic genes” and “monocistronic genes,” respectively. “up” and “down” represent “polycistronic upstream genes” and “polycistronic downstream genes,” respectively. “****” significant difference, *T*-test, *P* < 2.2e − 16

It is widely accepted that eukaryotes are not equipped with polycistrons, while, during gene curation, 1272 loci were found to be transcribed into single molecules overlapping with two or more PCGs, as supported by Iso-Seq transcripts (Fig. 6A). These loci were termed as polycistronic genes. Alternatively, PCGs within these loci could be transcribed independently, as evidenced by Iso-Seq transcripts and proteomics data. Collectively, the polycistronic genes were associated with 2815 PCGs. Specifically, 1011 polycistronic loci overlapped with two PCGs, 235 with three PCGs, and 22 with four PCGs. The lengths of polycistronic mRNAs ranged from 1028 to 10,459 bp, with an average of 3760.17 bp. The average protein length of PCGs within polycistronic genes (379.11 aa) was significantly shorter than that of monocistronic genes (449.86 aa) (Fig. 6B). Polycistronic gene pairs (average distance of 433.98 bp) were found to be significantly closer to each other compared to other adjacent gene pairs (average distance of 1356.41 bp) (Fig. 6C). In the linker regions of polycistrons that connecting individual genes, we found that some motifs occur frequently. For example, the TA-rich motif TACTTAYA was present in 14.6% of the analyzed sequences, while the GC-rich motif CGCCGCCGCCGYYC appeared in 13.2% of the sequences (Fig. 6D). Opal stop codons (TGA) were employed in nearly half of all the genes, with only minor differences observed between polycistronic genes and monocistronic genes (Fig. 6E). And we found that ~ 1/3 of the polycistronic genes were in frame when the relative reading frames of the upstream and the downstream PCGs were assessed (Fig. 6F). Among the polycistronic overlapped PCGs, 1258 (44.69%) were functionally annotated with the PFAM database, with enrichment in F-box-like and P450 domains (Fig. 6G). It is interesting that there were seven polycistronic genes consisted of two tandemly distributed P450 genes. Five polycistronic genes were randomly selected and confirmed by PCR amplification and Sanger sequencing (Fig. 6H and Additional file 1: Table S11).

## Discussion

A golden reference genome, manifested in complete and contiguous genome assembly and accurate gene models, can facilitate biological research greatly. With advances of sequencing technologies and assembly tools, high-quality genome assemblies for various organisms have emerged. Several tools for gene model prediction are available, including AUGUSTUS [29], GeneMark [30], MAKER [31], and EuGene [25]. Gene annotation typically relies on existing gene models; however, many genes within extensive gene pools remain underexplored, leading to potential inaccuracies in previously annotated genes. In addition, many genes are genetically different within species or even individuals, so it is difficult to guarantee the accuracy of gene annotation. Automatic annotation algorithms do not yet correctly identify all genes within a genome, and manual annotation is often necessary to obtain accurate gene models and gene sets. Due to the time-consuming nature of manual annotation, only a limited fraction of gene models in a genome was typically manually annotated. In recent years, manual correction of large-scale or genome-wide gene sets has received increasing attention from researchers [32]. Several high-quality genomes of *Ganoderma* species have been released [5], and the manually corrected gene set obtained in this study can provide excellent evidence and training/validation set for gene prediction of these genomes.

In this study, we manually corrected the whole genome gene sets of GL0102_53, utilizing Iso-Seq and RNA-Seq transcript data, achieving an ultra-high annotation quality for the *G. lingzhi* genome. In the gene correction process, the Iso-Seq data showed a greater advantage over the RNA-Seq data, with more accurate determination of gene structure, gene boundary, and 5′- and 3′-UTRs. However, Iso-Seq data were less effective than RNA-Seq data in recognizing very short exons, possibly due to the misalignment of short exons in long read alignment (for example, chr5g0072011, Additional file 1: Fig. S3). During the manual correction process, we identified numerous issues with the gene structures predicted by the software. Even for some well-characterized genes, such as P450, the software-dependent annotations are still prone to error. As a result, 83 novel genes were discovered during the manual correction process, indicating that the discovery of gene resources could benefit from meticulous gene examination. In addition, the corrected gene sets may serve for more accurate calculation of gene expression levels. These findings highlight the importance of high-quality genome annotation, which is essential for subsequent analyses, including evolutionary studies, developmental research, gene expression analysis, and variety identification. Although precise gene structures and annotations were obtained, limited to the current database, a large part of genes were annotated as “gene with unknown functions.” Future research on the functions of highly expressed or differentially expressed genes with unknown functions is crucial.

In this study, for the first time, the landscape of non-canonical splicing sites of *G. lingzhi* was deeply studied and a total of 94 types of non-canonical splicing sites were identified. Such a rich variety of non-canonical splicing sites increases the difficulty of gene annotation. GC-AG and AT-AC are two major non-canonical splice sites, which have been known for years, and genes were classified based on the presence/absence of non-canonical splice sites into four groups: GT-AG, GC-AG, AT-AC, and minor non-canonical splicing site genes [33], whereas in the present study, GC-AG (1.85%), GT-AC (0.053%), and GT-GG (0.041%) were the most abundant non-canonical splicing sites, with AT-AC comprising only 0.014%. These findings indicate a significant difference in the types and ratios of non-canonical splicing sites compared to those observed in plant and human genomes [11, 12].

To our knowledge, overlaps of coding regions among genes are common in viral genomes. However, validated gene overlaps had been documented in fungi. For example, in *Candida albicans*, the *CCT8* coding region overlaps 13 bp with the coding region of the convergently orientated *TRP1* gene [21]. And besides that, validated gene overlaps have been documented in mammals such as mice and humans [34, 35]. Herein, by manual correction, abundant overlapped genes were identified, indicating the simplicity of *G. lingzhi* genome. This reminds us that overlapped genes cannot be ignored or overlooked in future study of genome annotation. Unidirectional (i.e., same-strand) overlaps are most common in prokaryotes, while opposite or antiparallel-strand overlaps are more common in eukaryotes [34]. In this study, similar results were obtained for most overlapped pairs showed opposite gene transcription directions (different-strand overlaps). It is important to note that RNA-Seq data may struggle to accurately quantify these overlapped regions or genes if generated from short read, strand-nonspecific libraries. Only long read sequencing technologies can enable more precise quantification of such genes.

To date, comprehensive analyses of splice isoforms in filamentous fungi are lacking. In previous studies, AS was usually identified based on short-read RNA-Seq data. But short-read RNA-Seq data has disadvantages in identification of AS, as they are unsuitable for accurately reconstructing full-length splice isoforms. Recently, PacBio Iso-Seq has been employed to reveal the AS landscape in filamentous fungi [36]. In our study, we utilized full-length transcripts for AS detection, finding that 64.51% of genes in *G. lingzhi* undergo AS, which is significantly higher than previously reported ratios for fungi [20]. And more AS can be identified if more full-length transcripts were available, indicating an underestimation of AS ratio in macro-fungi. AS significantly increases *G. lingzhi* transcriptome complexity, expanding our view of the regulatory of RNA splicing in macro-fungi. Considering the importance of AS, it has been suggested that AS can be included as a standard analysis alongside gene expression analysis [37]. However, the involvement of AS in growth, development, phenotypic complexity, and environmental adaptability of *G. lingzhi* warrants further investigation.

Historically, it has been understood that each messenger RNA in eukaryotes encodes a single protein. Here, for the first time, abundant polycistronic genes were identified, underscoring their biological importance and enhancing our understanding of gene expression in *G. lingzhi*. What is the biological significance of the presence of polycistronic genes, whether it is a strategy for the regulation of gene expression, and the identification of the broad-spectrum intergenic signal that enables polycistronic expression require further experimental characterization. The co-expression of pairs within polycistronic transcripts in vitro may aid efforts to engineer *G. lingzhi* for research and industrial applications.

The maintenance of genetic variation is of adaptive significance [38]. In this study, a large number of genetic variations were identified among different strains of *G. lingzhi*, with many of these variations located within coding regions. The rich genetic resources and genotypic variations within this species contribute significantly to its phenotypic diversity and environmental adaptability.

Using the genome of *G. lingzhi* as an example, the complexity of fungal genomes exceeds our expectations. The high-quality gene annotation generated by extensive manual curation serves as the foundation for genomics-related studies of *G. lingzhi*. With the high-quality annotated genome and features of gene transcription, *G. lingzhi* can serve as a research model for other fungal species.

## Conclusions

In summary, a golden annotation with 14,147 high-confidence genes of *G. lingzhi* based on extensive manual correction was obtained. Novel characteristics of gene structure and gene transcription were also identified. Rich non-canonical splicing sites and genes with overlapped transcribed regions existed in the genome of *G. lingzhi*. More than 60% of *G. lingzhi* genes were alternatively spliced. And 1272 polycistronic genes which associated with 2815 PCGs were identified. The extraordinary gene structure and transcriptional activity identified by golden annotation can provide valuable insights for the study of medical fungi.

## Methods

### Strains, cultivation, and sample collection

The dikaryotic *G. lingzhi* strain GL0102 (the same strain with “Zhi 102” of Mycological Research Center, Fujian Agriculture and Forestry University) and two monokaryotic strains (GL0102_8 and GL0102_53) with opposite mating types, derived from it, were maintained on potato dextrose agar (PDA) at 4 °C. Besides, five monokaryotic *G. lingzhi* strains derived from protoplast monokaryogenesis were utilized in this study: GL0001_P5, GL0002_P2, GL0002_P3, GL0003_P1, GL0004_P6, and GL0005_P3. All strains were cultured at 28 °C on PDA plates.

Five-day-old mycelia (M) of GL0102 cultured on PDA plates were collected and quickly frozen in liquid nitrogen, while the remaining mycelia were inoculated into cultivation bags containing 1 kg of culture compost. The culture compost consisted of 10% oak wood, 70% sugarcane bagasse, 19% wheat bran, 1% gypsum, and a final water content of 60%. The bags were incubated in the dark at 28 °C with 50% ± 5% humidity and were transferred to the fruiting room once fully colonized by mycelia. In the fruiting room, the temperature was maintained at 28 °C ± 5 °C, room humidity at 85% ± 10%, with a 12-h light/dark cycle at 300 lx. Three replicates of Pe and Pl were collected and quickly frozen in liquid nitrogen.

### DNA extraction, genome sequencing, RNA-Seq and Iso-Seq

Liquid nitrogen-milled samples were subjected to DNA extraction using FineOut DNA kit (GENFINE Biotech (Beijing) CO., LTD, O301) following the manufacturer’s instructions. The purity, concentration, and integrity of the DNA were assessed using NanoDrop 8000, Qubit, and Femto Pulse. Genomic DNA (gDNA) with a concentration of ≥ 80 ng/μL, a total amount of ≥ 12 μg, and a primary band size of ≥ 30 kb on Femto Pulse was retained. For GL0102_8 and GL0102_53, 8 μg of gDNA was conjugated to a 16-bp barcode sequence, and then a 20 kb-insert-size library was constructed. The libraries were sequenced in one SMRT cell on the PacBio sequel II platform by Annoroad Gene Technology Co., Ltd, Beijing. In addition, 10 μg of gDNA from each of GL0102, GL0102_8, GL0102_53, GL0001_P5, GL0002_P2, GL0002_P3, GL0003_P1, GL0004_P6, and GL0005_P3 were used to construct paired-end libraries with an average insert size of 300 bp, sequenced on the Illumina NovaSeq platform by Annoroad Gene Technology Co., Ltd, Beijing.

M, Pe, and Pl samples were used for RNA-Seq and Iso-seq (RNA-Seq data for M was obtained from our previous study [28]). The total RNA extraction and quality-control of each sample were conducted using methods previously reported [39]. Briefly, liquid nitrogen-milled samples were subjected to RNA extraction using the RNA kit (OMEGA, R6827-01) following the manufacturer’s instructions, and RNA samples with an RNA Integrity Number ≥ 7.5 were retained. The RNA-Seq library construction and sequencing were carried out following protocols of MGI sequencing platform and at least 6 Gb of 150-bp paired-end reads were generated for each sample. The Iso-Seq libraries were constructed using SMRTbell prep kit following the manufacture’s protocols. Briefly, cDNA was synthesized and amplified, and SMRTbell libraries were constructed for each sample. All SMRTbell libraries were pooled and sequenced in one SMRT cell on the PacBio sequel II platform by Annoroad Gene Technology Co., Ltd, Beijing.

### Genome assembly

The quality of raw reads was assessed using FastQC (https://www.bioinformatics.babraham.ac.uk/projects/fastqc/) and SeqKit (https://bioinf.shenwei.me/seqkit/). Low-quality bases or reads were filtered out by Skewer [40] with the following criteria: trimming a 3′-end base to achieve quality > 30 and excluding reads with a length < 100 bp or an average quality < 30. The genome size was estimated by GenomeScope 2.0 [41]. PacBio data were assembled by Canu v1.8 [42] and polished with Racon [43] and Pilon [44]. The accuracy and completeness of the assembled genome were evaluated by K-mer analysis toolkit (KAT) [45], proovframe [46] and BUSCO analysis [47] with the fungi odb10 database.

### Repeat sequence

Dispersed repeated sequences at the DNA level were detected through an approach combining de novo prediction and homology-based searching. RepeatModeler v2.0.1 (http://www.repeatmasker.org/RepeatModeler/) was used to construct the de novo repeat library, and then the de novo library was mixed with Repbase (a database of eukaryotic repetitive elements) to conduct repeat searching using RepeatMasker v4.1.0 (http://www.repeatmasker.org/RMDownload.html).

### Gene annotation

The genomes of GL0102_8 and GL0102_53 were initially annotated using EuGene 4.2 [25]. Subsequently, the whole gene set of the GL0102_53 genome was manually corrected one-by-one in Apollo [26], based on alignments of Iso-Seq and RNA-Seq data with the genome sequence. Specifically, gene boundaries and splicing sites were adjusted according to the supporting RNA-Seq and Iso-Seq transcripts, utilizing a variety of operations, including deletion, mergence, split, creation, extension, retraction, and so on (Additional file 1: Fig. S5). Gffcompare v0.10.4 (https://ccb.jhu.edu/software/stringtie/gffcompare.shtml) was used to compare gene annotations before and after manual corrections. PCGs were functionally annotated by searching the following databases: Pfam [48], UniProt (https://sparql.uniprot.org/), eggNOG [49], and InterProScan [50].

### Identification of genetic variations among inter-strain and intra-strain of *G. lingzhi*

Eight genomes named after *G. lucidum* or *G. lingzhi* are available in the public database, of which five genomes shared over 95% similarity (based on the ITS2 fragment) with GL0102_53 (Additional file 1: Table S12). Besides, six re-sequenced *G. lingzhi* monokaryons (GL0001_P5, GL0002_P2, GL0002_P3, GL0003_P1, GL0004_P6, and GL0005_P3) were included in the genetic variation analysis. Genome-wide comparisons were conducted using Minimap2 (https://github.com/lh3/minimap2), with GL0102_53 as the reference, and variants were called using paftools. SNPs, indels (length shorter than 50 bp), and SVs (greater than or equal to 50 bp) were identified.

### Identification of AS and polycistronic genes

Full-length transcripts were generated from raw Iso-Seq reads by IsoSeq pipeline (https://github.com/PacificBiosciences/IsoSeq). The identification of whole-genome AS of GL0102_53 was conducted using SQANTI3 [51] based on the full-length transcript data from GL0102 across M, Pe, and Pl. The classification categories were as follows: Full splice match (matches all splicing junction perfectly), Incomplete splice match (matches the reference splicing junctions partially), Novel in catalog (novel isoform with a new combination of known splicing sites), Novel not in catalog (novel isoform with at least a new splicing site), Antisense (anti-sense to an annotated gene), Genic intron (within an intron), Genic genomic (overlaps introns and exons), and Intergenic (in the intergenic region). Polycistronic genes, which overlap two or more PCGs, were manually created in Apollo [26] based on full-length transcript data. Motifs enriched in linker regions of the polycistronic genes were identified using MEME [52].

### Validation of non-canonical splicing sites, overlapped genes, genetic variations, AS, and polycistronic genes

Regions containing non-canonical splicing sites, overlapped genes, genetic variations, AS, and polycistronic genes were randomly selected for analysis. Eighteen pairs of primers, designed according to sequences flanking the non-canonical splicing sites, were used to amplify a total of 24 non-canonical splicing sites, classified into 10 types (Additional file 1: Table S8). Four primer pairs, each based on the sequences of the two genes in the overlapped regions, were employed to amplify two pairs of overlapped genes (Additional file 1: Table S13). A total of 23 pairs of primers, designed according to conserved flanking regions, enabled the amplification of 59 SNPs, 24 indels, and 8 SVs (Additional file 1: Table S9). Additionally, 11 pairs of primers, derived from conserved regions, were used to amplify 11 genes exhibiting AS (Additional file 1: Table S10). Five pairs of primers, designed based on the sequences of upstream and downstream PCGs, facilitated the amplification of five polycistronic genes (Additional file 1: Table S11). The PCR amplification mixture consisted of 1 μL of cDNA or gDNA, 10 μL of 2 × Taq Master Mix (Vazyme Biotech Co., Ltd), 0.4 μL of 10 μmol/L forward and reverse primers, and 8.2 μL of ddH_2_O. The PCR reactions included an initial denaturation step at 95 °C for 3 min, followed by 34 cycles: 30 s at 95 °C, 30 s at 55–60 °C, and 30–90 s at 72 °C, concluding with a holding step at 72 °C for 5 min. For the detection of non-canonical splicing sites, amplifications using both gDNA and cDNA of GL0102 as templates were conducted. In contrast, for amplifying overlapped genes, AS, and polycistronic genes, only the cDNA of GL0102 was used as a template, while gDNA of GL0102_53 and GL0102_8 served as templates for detecting genetic variations. All PCR amplification products were analyzed via agarose gel electrophoresis and subsequently sequenced by Guangzhou IGE Biotechnology Co., Ltd.

### Western blot

Protein extracts from M was prepared using the Total Protein Extraction Kit (Solarbio, EX1100), following the manufacturer’s instructions. The protein concentration was detected using the Bradford Protein Assay Kit (Solarbio, PC0010), and 8 μg of protein was loaded onto a 10% SDS-PAGE gel. Following electrophoresis, the proteins were transferred to a polyvinylidene difluoride membrane, and western blot analysis was performed using antibodies against Gllac7, in accordance with the immunoblot protocol.

### Gene functional enrichment analysis

Pfam domains and GO enrichment analyses were carried out using the clusterProfiler [53], and enrichment results with *p*-value < 1e − 3 were retained.

### Proteomics

The proteomic libraries utilized in this study were prepared from trypsin-digested total protein extracts derived from pools of M, Pe, and Pl. The digested peptides were separated into six fractions with Pierce high pH reversed-phase fractionation kit (Thermo scientific). And then LC–MS/MS analysis was performed on a Q Exactive mass spectrometer (Thermo Scientific). The proteomics data were analyzed with MaxQuant v2.4.14.0, and 11,161 peptides corresponding to 2826 proteins were identified.

## Supplementary Information

Additional file 1: Fig. S1-S5. Table S1-S13. Fig. S1 *K*-mer spectrum analysis. Fig. S2 Gene structures of two P450 genes before and after correction. Fig. S3 Non-canonical splicing sites. Fig. S4 Agarose gel electrophoresis of PCR amplification products of alternative splicing genes. Fig. S5 Nine typical scenarios of manually gene correction with Apollo. Table S1 Statistics of Illumina and PacBio data. Table S2 Assemblies of GL0102_8 and GL0102_53. Table S3 Statistics of the repeat elements of the GL0102_8 and GL0102_53 genomes. Table S4 BUSCO assessment results of GL0102_53 genome. Table S5 Statistics of RNA-Seq and Iso-Seq data of M, Pe, and Pl. Table S6 Mapping rates of gene matching in public databases. Table S7 Donor and acceptor of non-canonical splicing sites. Table S8 Primers used in validation of non-canonical splicing sites. Table S9 Primers used in validation of genetic variations between GL0102_8 and GL0102_53. Table S10 Primers used in validation of alternative splicing events. Table S11 Primers used in validation of polycistronic genes. Table S12 Genomes of *G. lingzhi* used in genetic variation analysis. Table S13 Primers used in validation of overlapped genes.

Additional file 2: Fig. S6 Uncropped blots for Fig. 5E of anti-Gllac7.

## Data Availability

All data related to genome sequencing, genome assembly, genome annotation, RNA-Seq, and Iso-Seq reported in this study have been deposited in the NCBI and are available under PRJNA1177388 [54]. Additionally, this data can also be accessed through GPGD (http://www.gpgenome.com/species/408). The RNA-Seq data of M used in this have been deposited in GPGD [55]. The genome assemblies of *G. lingzhi* (*G. lucidum*) used in this study are available in NCBI under the following accession numbers: GCA_000271565.1 [56], GCA_000338035.1 [57], GCA_019426095.1 [58], GCA_026283605.1 [59], and GCA_033032785.1 [60].

## Abbreviations

AS: Alternative splicing
BUSCO: Benchmarking Universal Single-Copy Ortholog
Pe: Early primordia
GO: Gene Ontology
Indels: Insertion/deletions
Iso-Seq: Isoform sequencing
Pl: Late primordia
M: Mycelia
PCGs: Protein-coding genes
RNA-Seq: RNA sequencing
SNPs: Single-nucleotide polymorphisms
SVs: Structural variations
