# Supplementary material for: The golden genome annotation of Ganoderma lingzhi reveals a more complex scenario of eukaryotic gene structure and transcription activity
Source: BMC Biol. 2024 Nov 25;22:271. doi: 10.1186/s12915-024-02073-y (PMC11590231; doi:10.1186/s12915-024-02073-y)
Supplement: Supplementary file 1 — Additional file 1: Fig. S1-S5. Table S1-S13. Fig. S1 K-mer spectrum analysis. Fig. S2 Gene structures of two P450 genes before and after correction. Fig. S3 Non-canonical splicing sites. Fig. S4 Agarose gel electrophoresis of PCR amplification products of alternative splicing genes. Fig. S5 Nine typical scenarios of manually gene correction with Apollo. Table S1 Statistics of Illumina and PacBio data. Table S2 Assemblies of GL0102_8 and GL0102_53. Table S3 Statistics of the repeat elements of the GL0102_8 and GL0102_53 genomes. Table S4 BUSCO assessment results of GL0102_53 genome. Table S5 Statistics of RNA-Seq and Iso-Seq data of M, Pe, and Pl. Table S6 Mapping rates of gene matching in public databases. Table S7 Donor and acceptor of non-canonical splicing sites. Table S8 Primers used in validation of non-canonical splicing sites. Table S9 Primers used in validation of genetic variations between GL0102_8 and GL0102_53. Table S10 Primers used in validation of alternative splicing events. Table S11 Primers used in validation of polycistronic genes. Table S12 Genomes of G. lingzhi used in genetic variation analysis. Table S13 Primers used in validation of overlapped genes. [file 12915_2024_2073_MOESM1_ESM.docx]

Table S1 Statistics of Illumina and PacBio data

| **Illumina PE150 sequencing** |  |  |  |  |  |  |  |  |
| --- | --- | --- | --- | --- | --- | --- | --- | --- |
| **Library ID** | **Number of reads** | **Total data** | **GC content** | **Q20 (%)** | **Q30 (%)** |  |  |  |
| GL0102 | 30,971,330 | 4,645,699,500 | 53.8137 | 98.749 | 96.6315 |  |  |  |
| GL0102_53 | 31,019,780 | 4,652,967,000 | 53.2071 | 98.7638 | 96.7018 |  |  |  |
| GL0102_8 | 33,490,090 | 5,023,513,500 | 52.2904 | 97.9068 | 95.1192 |  |  |  |
|  |  |  |  |  |  |  |  |  |
| **PacBio Sequel II long reads** |  |  |  |  |  |  |  |  |
| **Library ID** | **Number of reads** | **Total data** | **GC content** | **Average length** | **N50** | **Q20 (%)** | **Q30 (%)** | **Average Q-score** |
| GL0102_53 | 612,143 | 7,307,587,854 | 45.7202 | 11,937.7 | 14,285 | 94.51 | 87.22 | 24.17 |
| GL0102_8 | 825,036 | 11,547,862,484 | 48.0095 | 13,996.8 | 16,662 | 93.73 | 85.30 | 23.03 |

Table S2 Assemblies of GL0102_8 and GL0102_53

|  | **GL0102_53** | | **GL0102_8** | |
| --- | --- | --- | --- | --- |
|  | **Contig** | **Chromosome** | **Contig** | **Chromosome** |
| Total sequences | 146 | 13 | 74 | 13 |
| Total bases | 50,763,784 | 48,562,602 | 47,112,404 | 46,347,626 |
| Average sequence length | 347,697.2 | 3,735,584.77 | 636,654.1 | 3,565,202 |
| N50 | 1,609,567 | 4,668,029 | 3,188,751 | 4,968,520 |
| N90 | 321,659 | 2,645,415 | 502,496 | 2,471,032 |
| (G + C)s | 55.73% | 55.94% | 55.89% | 56.01% |
| Concordance* | 99.49% | 99.44% | 99.46% | 99.44% |

“*” represent concordance between assembly and long reads calculated by proovframe

Table S3 Statistics of the repeat elements of the GL0102_8 and GL0102_53 genomes

| **Type** | | | **GL0102_8** | | | | | |  | | **GL0102_53** | | | | | |  |
| --- | --- | --- | --- | --- | --- | --- | --- | --- | --- | --- | --- | --- | --- | --- | --- | --- | --- |
|  |  |  | **#elements** | | **Length (bp)** | | **%genome** | |  | | **#elements** | | **length(bp)** | | **%genome** | |  |
| Retroelements |  |  | | 1687 | | 2737069 | | 5.81 | |  | | 2165 | | 4900465 | | 9.65 | |
|  | SINEs | | 28 | | 2079 | | 0.00 | |  | | 14 | | 728 | | 0.00 | |  |
|  | LINEs | | 247 | | 137535 | | 0.29 | |  | | 222 | | 256523 | | 0.51 | |  |
|  | LTR elements | | 1412 | | 2597455 | | 5.51 | |  | | 1929 | | 4643214 | | 9.15 | |  |
| DNA transposons |  | | 545 | | 480441 | | 1.02 | |  | | 755 | | 1276989 | | 2.52 | |  |
| Rolling-circles |  | | 23 | | 3978 | | 0.01 | |  | | 40 | | 8000 | | 0.02 | |  |
| Unclassified |  | | 3999 | | 2646524 | | 5.62 | |  | | 4225 | | 2467918 | | 4.86 | |  |
| Total interspersed repeats |  | |  | | 5864034 | | 12.45 | |  | |  | | 8645372 | | 17.03 | |  |
| Small RNA |  | | 221 | | 97582 | | 0.21 | |  | | 202 | | 79531 | | 0.16 | |  |
| Satellites |  | | 45 | | 6529 | | 0.01 | |  | | 43 | | 8920 | | 0.02 | |  |
| Simple repeats |  | | 7351 | | 313969 | | 0.67 | |  | | 7406 | | 326414 | | 0.64 | |  |
| Low complexity |  | | 1060 | | 54523 | | 0.12 | |  | | 1040 | | 52373 | | 0.10 | |  |

Table S4 BUSCO assessment results of GL0102_53 genome

|  |  | Original | Corrected |
| --- | --- | --- | --- |
| Fungi_odb10 (total 758) | Complete | 680 (89.7%) | 751 (99.1%) |
|  | Complete & single copy | 666 (87.9%) | 689 (90.9%) |
|  | Complete & duplicated | 14 (1.8%) | 62 (8.2%) |
|  | Fragmented | 26 (3.4%) | 3 (0.4%) |
|  | Missing | 52 (6.9%) | 4 (0.5%) |
|  |  |  |  |
| Agaricomycetes_odb10 (total 2898) | Complete | 2561 (88.4%) | 2840 (98%) |
|  | Complete & single copy | 2536 (87.5%) | 2558 (88.3%) |
|  | Complete & duplicated | 25 (0.9%) | 282 (9.7%) |
|  | Fragmented | 120 (4.1%) | 19 (0.7%) |
|  | Missing | 217 (7.5%) | 39 (1.3%) |
|  |  |  |  |
| Polyporales_odb10 (total 4464) | Complete | 3916 (87.7%) | 4326 (96.9%) |
|  | Complete & single copy | 3861 (86.5%) | 3833 (85.9%) |
|  | Complete & duplicated | 55 (1.2%) | 493 (11.0%) |
|  | Fragmented | 140 (3.1%) | 31 (0.7%) |
|  | Missing | 408 (9.2%) | 107 (2.4%) |

Table S5 Statistics of RNA-Seq and Iso-Seq data of M, Pe, and Pl

| **RNA-Seq** |  |  |  |  |  |
| --- | --- | --- | --- | --- | --- |
| **Library ID** | **Number of reads** | **Total data** | **GC content** | **Q20** | **Q30** |
| GL0102_Pe_1 | 44,809,898 | 6,721,484,700 | 59.1506 | 99.1453 | 97.4923 |
| GL0102_Pe_2 | 44,494,130 | 6,674,119,500 | 59.3069 | 99.1556 | 97.503 |
| GL0102_Pe_3 | 45,455,594 | 6,818,339,100 | 58.8482 | 99.1327 | 97.4594 |
| GL0102_Pl_1 | 47,961,786 | 7,194,267,900 | 59.4184 | 99.1443 | 97.5005 |
| GL0102_Pl_2 | 47,390,856 | 7,108,628,400 | 59.4667 | 99.1134 | 97.4505 |
| GL0102_Pl_3 | 46,083,180 | 6,912,477,000 | 59.0747 | 99.0509 | 97.2562 |
| GL0102_M_1 | 44,873,064 | 6,730,959,600 | 59.6648 | 98.9224 | 97.0007 |
| GL0102_M_2 | 40,470,614 | 6,070,592,100 | 59.5293 | 98.8887 | 96.9823 |
| GL0102_M_3 | 47,992,420 | 7,198,863,000 | 59.6119 | 98.8065 | 96.7515 |
|  |  |  |  |  |  |
| **Iso-Seq** |  |  |  |  |  |
| **Library ID** | **Number of reads** | **Total data** | **GC content** | **Average length** | **N50** |
| GL0102_M | 892,979 | 2,356,603,945 | 58.0638 | 2,639 | 2,919 |
| GL0102_Pe | 878,303 | 1,879,138,873 | 57.3788 | 2,139.5 | 2,442 |
| GL0102_Pl | 866,293 | 1,972,356,095 | 57.3933 | 2,276.8 | 2,605 |
|  |  |  |  |  |  |

Table S6 Mapping rates of gene matching in public databases

| **Database** | **Original** | **Corrected** |
| --- | --- | --- |
| Pfam | 39.47% | 52.7% |
| EggNOG | 59.11% | 72.63% |
| UniProt | 37.38% | 49.91% |

Table S7 Donor and acceptor of non-canonical splicing sites

| **Donor** | | | **Acceptor** | | |
| --- | --- | --- | --- | --- | --- |
| **Type** | **Number** | **Ratio (%)** | **Type** | **Number** | **Ratio (%)** |
| GT | 90,139 | 97.8633546 | AG | 91,729 | 99.5896077 |
| GC | 1,711 | 1.85762211 | AC | 71 | 0.07708426 |
| GA | 38 | 0.04125636 | GG | 47 | 0.05102761 |
| AT | 37 | 0.04017067 | TG | 30 | 0.03257081 |
| CA | 31 | 0.03365651 | CG | 28 | 0.03039943 |
| CT | 29 | 0.03148512 | CC | 27 | 0.02931373 |
| GG | 25 | 0.02714235 | AA | 27 | 0.02931373 |
| CG | 14 | 0.01519971 | GC | 26 | 0.02822804 |
| CC | 14 | 0.01519971 | GT | 20 | 0.02171388 |
| TC | 13 | 0.01411402 | AT | 20 | 0.02171388 |
| AG | 13 | 0.01411402 | CT | 17 | 0.01845679 |
| TA | 12 | 0.01302833 | TC | 16 | 0.0173711 |
| AC | 10 | 0.01085694 | TA | 16 | 0.0173711 |
| TG | 8 | 0.00868555 | TT | 14 | 0.01519971 |
| TT | 7 | 0.00759986 | GA | 13 | 0.01411402 |
| AA | 6 | 0.00651416 | CA | 6 | 0.00651416 |

Table S8 Primers used in validation of non-canonical splicing sites

| **Type** | **Loci** | **Forward (5’-3’)** | **Reverse (5’-3’)** | **Amplification length in cDNA (bp)** | **Amplification length in gDNA (bp)** | **Note** |
| --- | --- | --- | --- | --- | --- | --- |
| GC-AG | chr3g0037881 | CTCCTCTATCTCCTCCTGAA | CTGATCTGCTTCCGTCTC | 399 | 525 |  |
| GC-AG | chr3g0044481 | GTCCGATCAGCAAGAAGTA | AACAGAACGCATCCAAGAT | 480 | 648 |  |
| GC-AG | chr1g0010561 | GTCAACACCGTCCTCATC | GAACTCCTTCGCTACTTGG | 249 | 358 |  |
| AT-AC | chr1g0010881 | AAGGAGGATGCCGACAGGA | TCGTTGAGTGGCTTGAAGTTC | 398 | 536 |  |
| AA-AC | chr3g0037891 | CACAATCCTCTCAGCACTAT | CGAAGACGATGGACAGAAT | 344 | 368 |  |
| GT-CT | chr3g0043651 | CTTGGTGATGGAGGTGTAG | CTCTTGTCCTGCCGTATC | 162 | 341 |  |
| TG-AT | chr3g0037041 | ATCCGAAGGTTTGCCAAG | TGTATGCTCCAGACAAGTG | 147 | 1,101 | Failed |
| GT-GG | chr5g0065591 | CGCAGTATCGCTGTCATT | TTGGACCGTACTCGTTGA | 368 | 441 |  |
| CC-CT | chr5g0067211 | TCACTCGCTCAACTTCTG | GCTCTTCCTTCTACCACAA | 1,121 | 1,504 |  |
| GC-AG | chr5g0071881 | TACCGACGACCTACCTTC | CTTCTCCACCGCAATAGTT | 1,325 | 1,666 | Two GC-AG |
| GC-AG | chr5g0072011 | ATCCTCAGATTCCGAAGAAC | AGTCATCATCAGCGTCATC | 1,749 | 2,432 | Three GC-AG |
| GT-TC | chr6g0083751 | AATTGTGCGTGCCGTACTAC | GTTCTGCTGATGATCGTGACTC | 641 | 1,115 | Failed |
| AT-AC | chr6g0094801 | CTCACATCACCGACTTCC | CATCCTCCTTCTGCTCAC | 1,026 | 1,368 | AT-AC and GC-AG |
| CC-TA | chr6g0096681 | AAGAGGCAACACCGAATG | GGCAACTGGCTGATTAGG | 767 | 815 |  |
| GC-AG | chr7g0114351 | GTCAGCCTTACTCTGTCTC | AGATACGGAACGGAACATC | 613 | 913 | Two GC-AG |
| GT-TA | chr7g0117171 | CCTCTTGCTCACATCTATCA | CCTCGTCAATCGTCTCTG | 652 | 952 | GT-TA and GC-AG |
| GT-AT | chr7g0121191 | AACTGAACACCGCATACTT | CTTCTTGGCTGAGGAACTT | 366 | 520 |  |
| TC-AG | chr7g0128491 | CTCAACAACAGCGGATACA | AACGACGGTACATCACAATA | 608 | 747 |  |

Table S9 Primers used in validation of genetic variations between GL0102_8 and GL0102_53

| **Name** | **Forward (5’-3’)** | **Reverse (5’-3’)** | **Length in 8/ 53 (bp)** | **Number of loci that can be validated** |
| --- | --- | --- | --- | --- |
| SNP_1 | CACCTCCAATACACATCAGT | ACGAATCACCACCAGAGA | 470/470 | 18 |
| SNP_2 | TAGCACATTCATCCTCGC | CAGCTTCTTGTTCGTGTTG | 438/438 | 5 |
| SNP_3 | ATGGCGMAGAAGGAGAAG | CGAGTCAAGCAGGATCTC | 225/225 | 5 |
| SNP_4 | CAGATGAGAGGYCGTGTC | TTGGATGTGAGGCAAYGG | 216/216 | 13 |
| SNP_5 | AAGATGCGGAAGCCTGAT | TGAACACCACCGAACTCT | 254/254 | 3 |
| SNP_6 | CCGTTGTCCTGTTGTTGA | AGTATYCACCGTCTCGTATA | 993/993 | 15 |
| Indel_1 | CATCGCTGGTCTGAGTAA | CTGCCATACACGAGGTAG | 246/251 | 1 |
| Indel_2 | CGCCTATCAGCAAGCATT | CGGGAAGAGTTGTGTAGC | 146/153 | 1 |
| Indel_3 | TGGACAGAGACAGTAAGAAG | CATACAGTCAAGTGGTCAAC | 629/635 | 1 |
| Indel_4 | CATATTCCATTGCTGCTCTG | CTCTTCTACCTCCTCATCCT | 821/776 | 1 |
| Indel_5 | CCATTCTACCGCCAACAT | CAACGACTTCAGCAACAC | 1364/1283 | Five indels with length of 5, 24, 13, 36, and 3 bp |
| Indel_5 | TCTCGTAGTATCTGCCAATG | CGGATAGTTAGGACCTTCAG | 250/283 | 1 |
| Indel_6 | GGCRAGTGAGGTGATAGC | AGTGGYGGTGGATTCMGAT | 572/598 | Two indels with length of 4 and 22 bp |
| Indel_7 | ATCGACGAAGGTAAGACTG | AGACGGTAGGAGGAYAGC | 292/312 | 1 |
| Indel_8 | CACAGGTCCGTTGATTGG | ATCGCTGCTCACTATCGT | 1454/1473 | Three indels with length of 10, 7, and 2 bp |
| Indel_9 | ACTGTTGTAGGAGAGGAGAT | ACGGTGGAATGAGCAATG | 614/626 | Two indels with length of 8 and 4 bp |
| SV_1 | GAGCAGACAGTGATGAGAT | CTTCGGAGACAAGACCTATA | 875/385 | 1 |
| SV_2 | GTGAAGAACTTGAGGAGGAA | CGATGGTGTGACTAGAGTG | 541/1764 | 1 |
| SV_3 | GGATGCTGGTGAAGAAGG | TCGTGTCGTCAGTTATGTAG | 306/890 | 1 |
| SV_4 | CTGACAGGTTCGTAGAYGAT | GAGTGACCATTGAGGAAGT | 542/1009 | 1 |
| SV_5 | TCCATAGTTCACYTGTTCTC | GACGACCGAGCATACTTAT | 447/895 | An SV with length of 449 bp, four indels with length of 4, 11 ,6, and 10 bp |
| SV_6 | TGATAGACAGGCAGACAGA | AGACGCTCAGATAACTTAGG | 774/391 | 1 |
| SV_7 | CAAGATGCGTGAGAACAAG | AGCGTGGTWGTGATGGAT | 421/685 | An SV with length of 254 bp, an indel with length of 10 bp |
| SV_8 | GGTCTTGATTGCCTTAGTATAG | ACGAGGTGTTCCATTGTG | 1141/1394 | 1 |

Note: Indel_5 and SV_8 showed incorrect amplification, ‘8’ and ‘53’ represent GL0102_8 and GL0102_53, respectively.

Table S10 Primers used in validation of alternative splicing events

| **Name** | **Forward (5’-3’)** | **Reverse (5’-3’)** | **Amplification length (bp)** | **Annotation** | **Catalog of alternative splicing** |
| --- | --- | --- | --- | --- | --- |
| chr3g0049171 | AGACCTGACCTGAATACGA | AAGGCATATAAGCAGTGTGA | 1,841/2,089 | Galactose oxidase | Novel Not in Catalog, new donor |
| chr1g0004681 | CATCTTCGTTCACCTCCA | AATAAGCACAACACCAACC | 704/790 | Opy2 protein | Incomplete Splice Match, intron retention |
| chr3g0047961 | CGACGATGAGGACAACTG | GTCAGCCTAGCCAACTTG | 630/691 | Glycosyltransferase Family 8 protein | Incomplete Splice Match, intron retention |
| chr3g0055031 | CCAACAGTCGCAACAATG | ATTCATCGCCGTGATAGC | 429/980 | RNA-binding domain-containing protein | Incomplete Splice Match, intron retention |
| chr3g0055201 | TTATGCTGGAGCCTGTTG | TATCACGACGACCTTCTTG | 341/388 | Alpha/beta-hydrolase | Incomplete Splice Match, intron retention |
| chr1g0015631a | GGAGCATGTTGAGCAGAT | CTATTGACGGATGGTTGATAC | 257/314 | Pkinase-domain-containing protein | Novel In Catalog, combination of known splice sites |
| chr1g0017881 | GCGACTTCCTCTTCTACTC | TTCTGTCCACTCAACTTCC | 641/760 | PRTase-like protein | Incomplete Splice Match, intron retention |
| chr2g0025801 | CACCATCTCTTCCAATCTCT | CTCGCTGATAGGCACTTC | 829/999 | GMC oxidoreductase | Novel Not in Catalog, new donor |
| chr4g0063901a | GGCTCCGTGTTCTCATAC | ATAACGCATAGGCAGATTCA | 579/644 | RNA-binding domain-containing protein | Novel Not in Catalog, new donor |
| chr5g0070341 | CTGGAACACTACCGATGATA | ACAACGAGTAAGATGACACA | 748/787 | RNA-binding domain-containing protein | Novel In Catalog, combination of known splice sites |
| chr5g0077891 | GTTCAACACGCTCTCCAA | ATTCCAATAGTCACGGTCAT | 746/856 | Aspartyl protease | Novel Not in Catalog, new donor |

Note: chr3g0055031 showed incorrect amplification

Table S11 Primers used in validation of polycistronic genes

| **Name** | **Forward (5’-3’)** | **Reverse (5’-3’)** | **Length (bp)** | **Annotation of polycistronic genes** |
| --- | --- | --- | --- | --- |
| P1 | AACTACAGGAACGAGACTAC | GGAGATGGAGAGGCAATG | 3,610 | Unknown/unknown |
| P2 | GACCTCCGCACTACTACT | AGAACCGACTACGCTACA | 2,675 | P450/unknown |
| P3 | CTTGAAGGAGTGTGATGGT | GCGTGAAGGCAATTAGGA | 3,019 | MFS/Isocitrate dehydrogenase |
| P4 | CCATCGGAATCGGAATCAT | CAGCTTGACGAGTGTACC | 1,122 | Wax ester synthase/laccase |
| P5 | CCGTCGTCATCGTCCTCAA | CTCCACTCCATATAGAATCGCATT | 2,094 | Unknown/unknown |

Table S12 Genomes of *G. lingzhi* used in genetic variation analysis

| **Strain** | **GCA#** | **Submitter** | **Release date** |
| --- | --- | --- | --- |
| CGMCC5.0026 | GCA_000271565.1 | Institute of Medicinal Plant Development | 2012/06/21 |
| BCRC 37180 | GCA_000338035.1 | Ganoderma lucidum Research Consortium | 2013/02/01 |
| Ling-Jian No.2 | GCA_019426095.1 | South China Botanical Garden, Chinese Academy of Sciences | 2021/07/30 |
| SCIM1006 | GCA_026283605.1 | Central South University of Forestry & Technology | 2022/11/22 |
| IA20 | GCA_033032785.1 | Universidad EAFIT | 2023/10/25 |
| GL0004_P6 | - | This study | - |
| GL0003_P1 | - | This study | - |
| GL0001_P5 | - | This study | - |
| GL0005_P3 | - | This study | - |
| GL0002_P2 | - | This study | - |
| GL0002_P3 | - | This study | - |
| GL0102_8 |  | This study | - |
| GL0102_53 |  | This study | - |

Table S13 Primers used in validation of overlapped genes

| **Name** | **Forward (5’-3’)** | **Reverse (5’-3’)** | **Amplification length (bp)** |
| --- | --- | --- | --- |
| chr2g0025701c | ACAGCGGTTCCTAATACAAG | ACGACATATAGAGAAGCATCC | 713 |
| chr2g0025701a | TACTCCTCTTCGCCTCAA | ACCTGTATCCAGACATCATC | 333 |
| chr1g0000801 | CGTAGACGGAACTCTCATC | AGGATATGGAACGGACAATG | 468 |
| chr1g0000811 | TTCCAATCGCTCGGTTGTG | TCCTCGCCTCATCGTTCAA | 1,354 |


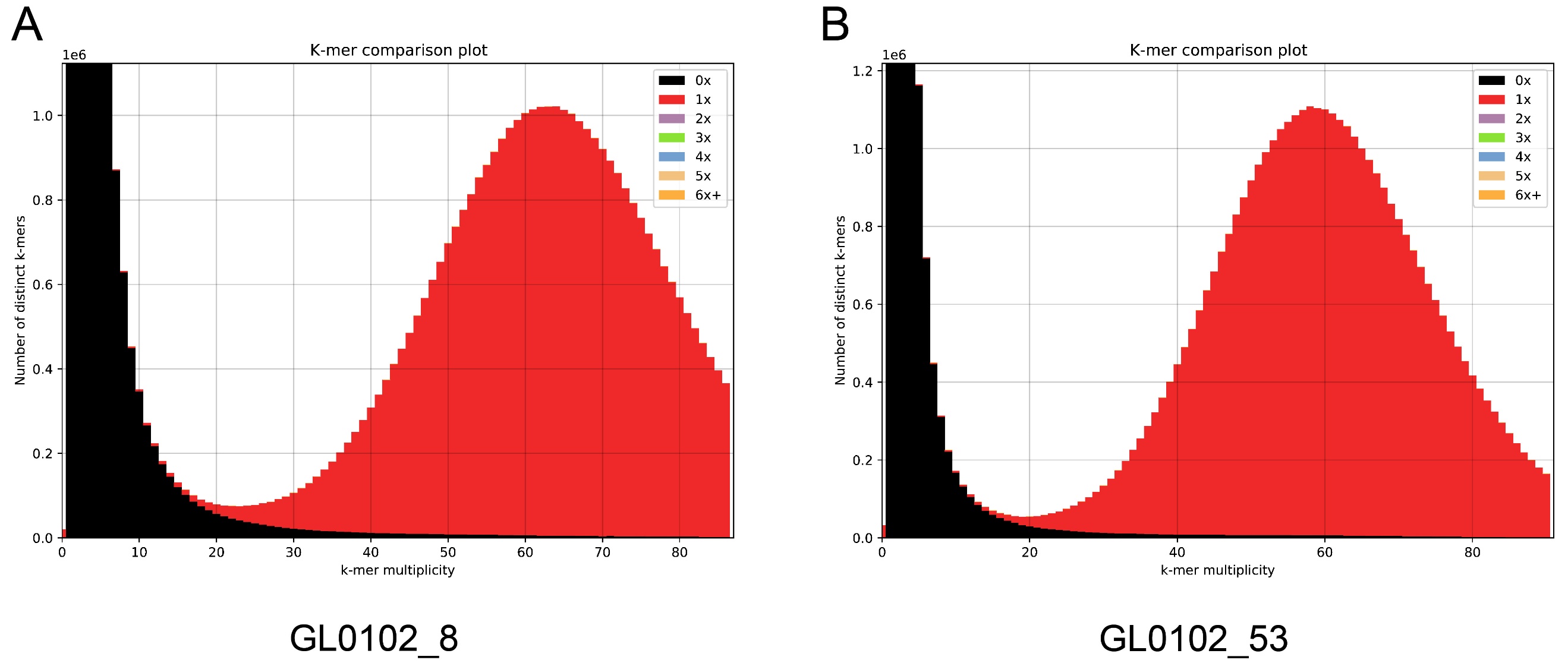


Fig. S1 *K*-mer spectrum analysis. Comparison of 27-mer between assembled contigs and long reads by the KAT program. 0x – 6x+ means *k*-mers of long reads could be found in contigs *k*-mers 0 – 6+ times. There was no peak of 0x indicating no fragment loss in the assembly.


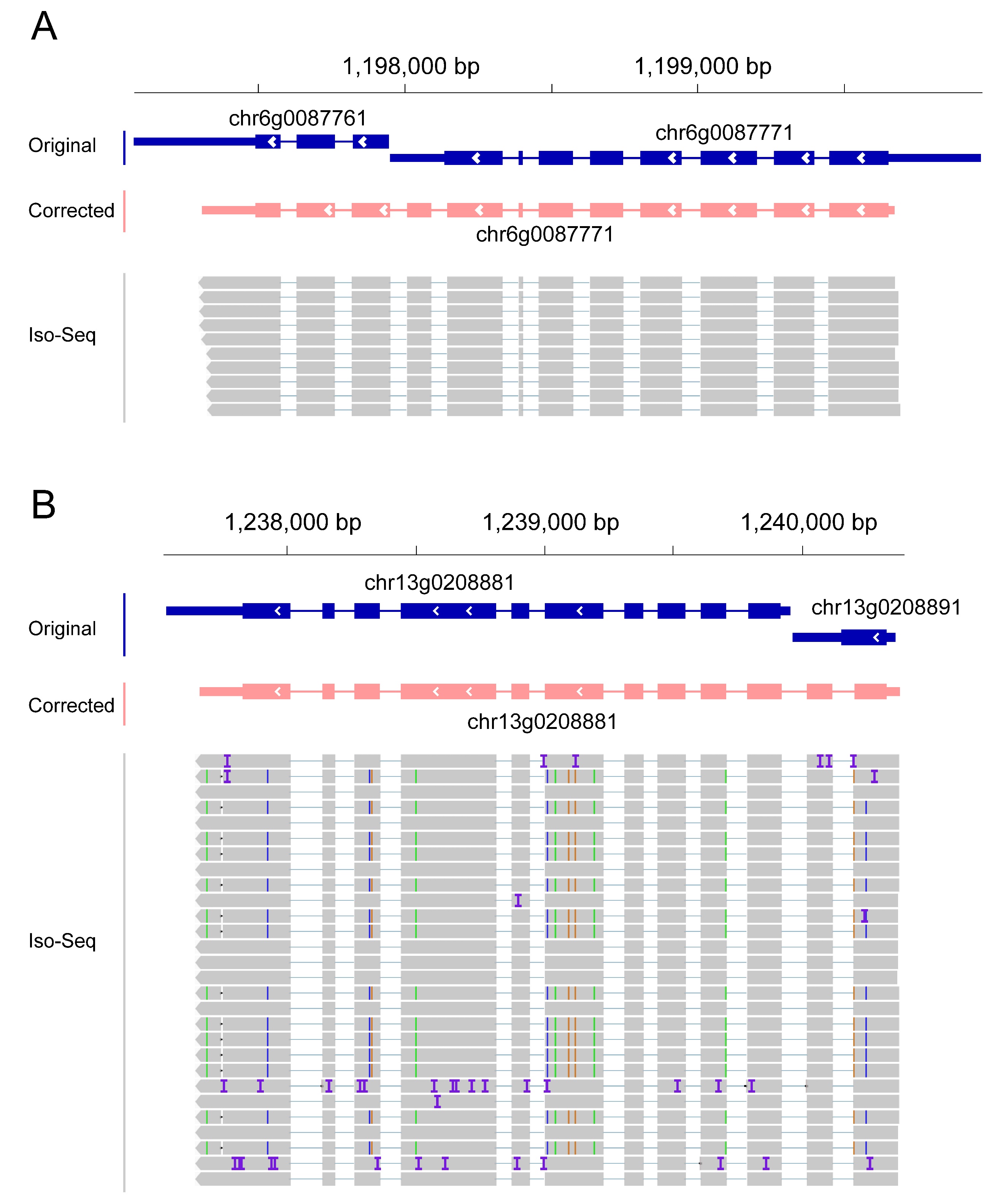


Fig. S2 Gene structures of two P450 genes before and after correction. Arrows indicate the direction of transcription.


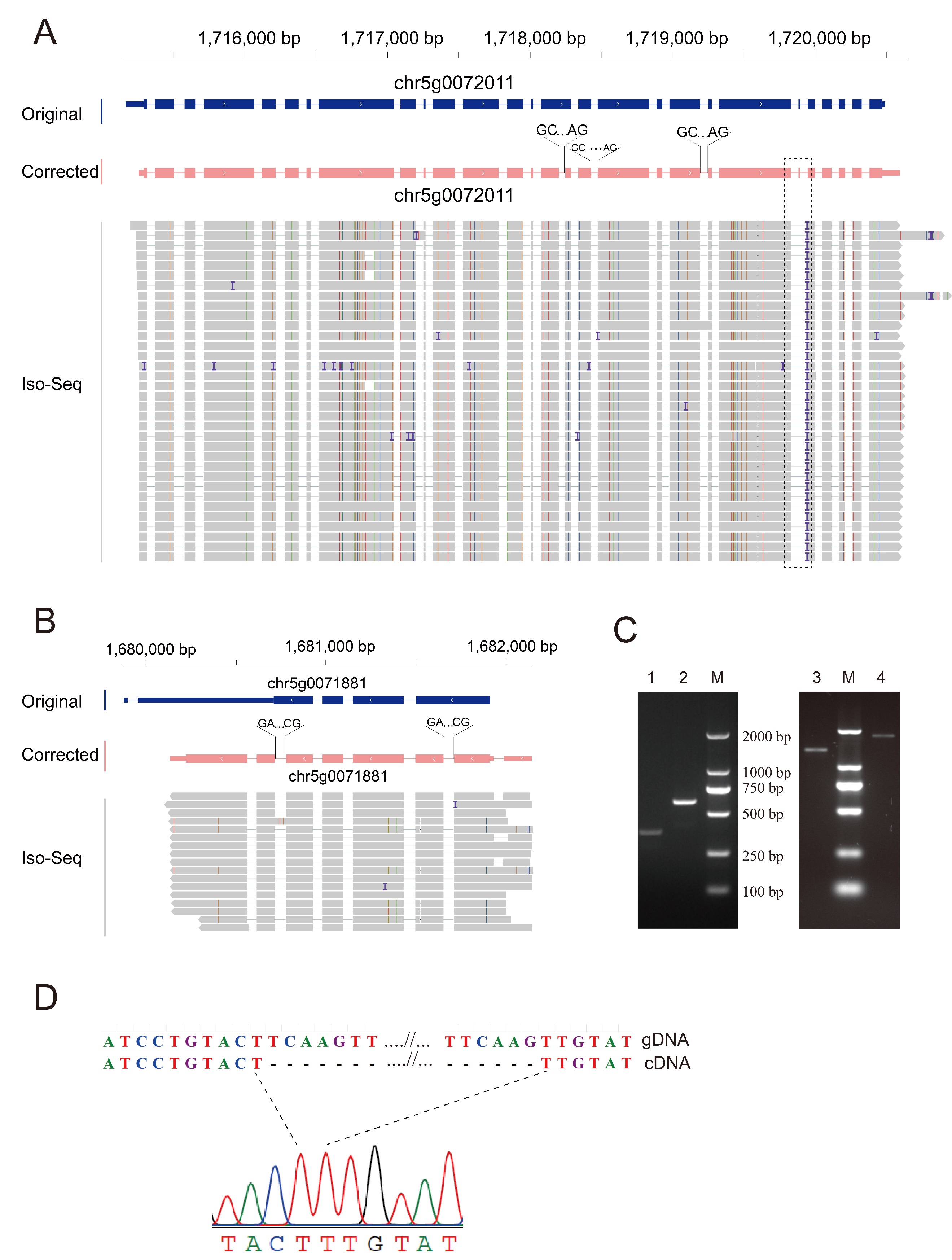


Fig. S3 Non-canonical splicing sites. (A) P450 genes containing three GC-AG splicing sites. Full-length transcripts are unfavorable for identifying extremely short exons (Shown in black dashed rectangular box). (B) P450 genes containing two GC-AG splicing sites. Arrows indicate the direction of transcription. (C) Agarose gel electrophoresis of PCR products containing non-canonical splicing sites. Lane 1-4 represent PCR amplification products containing GT-AT, TC-AG, two GC-AG, and three GC-AG splicing sites, respectively. (D) Sanger sequencing of products containing TC-AG splicing sites.


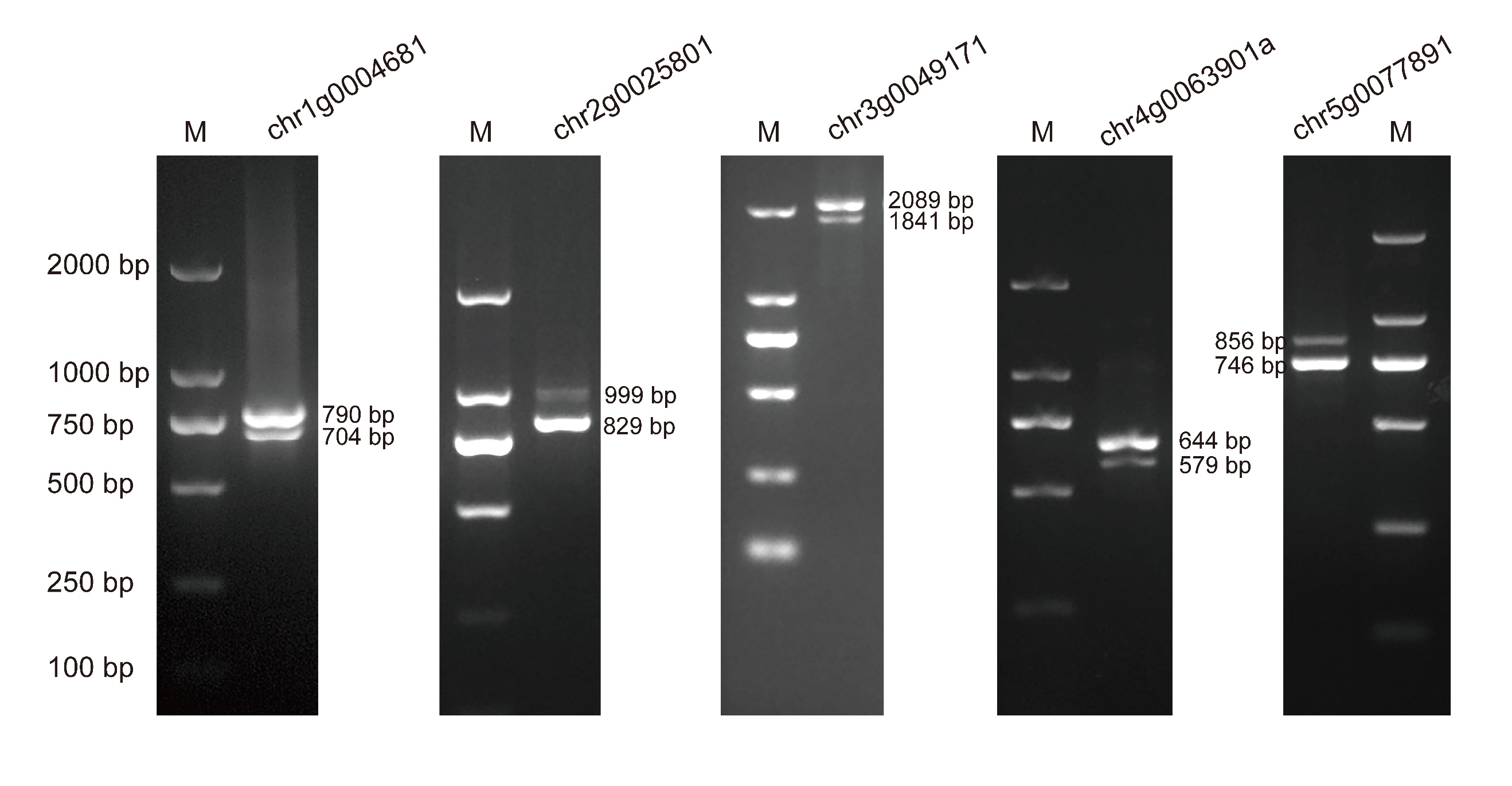


Fig. S4 Agarose gel electrophoresis of PCR amplification products of alternative splicing genes


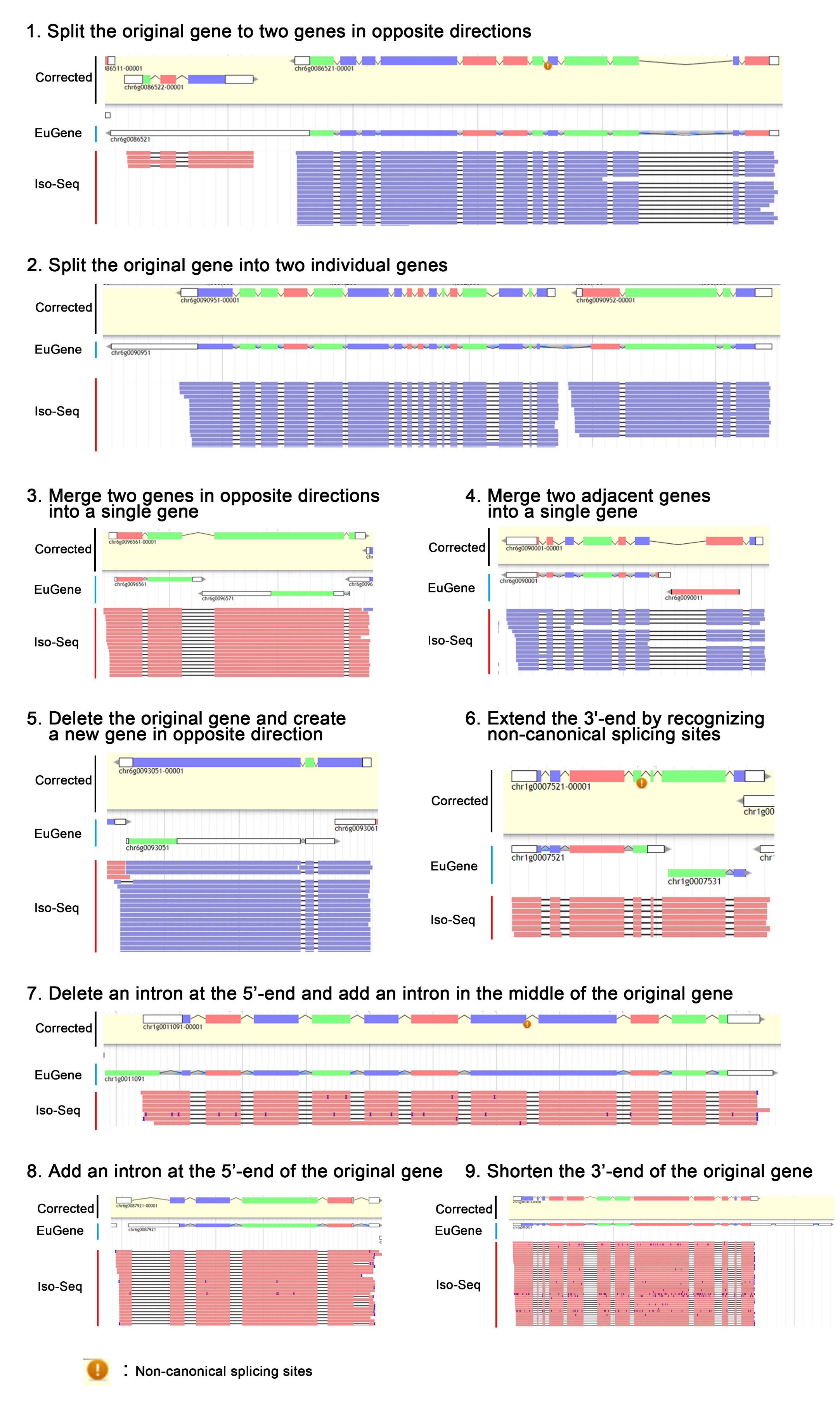


Fig. S5 Nine typical scenarios of manually gene correction with Apollo
