# Supplementary figures and images for: The golden genome annotation of Ganoderma lingzhi reveals a more complex scenario of eukaryotic gene structure and transcription activity
Source: BMC Biol. 2024 Nov 25;22:271. doi: 10.1186/s12915-024-02073-y (PMC11590231; doi:10.1186/s12915-024-02073-y)

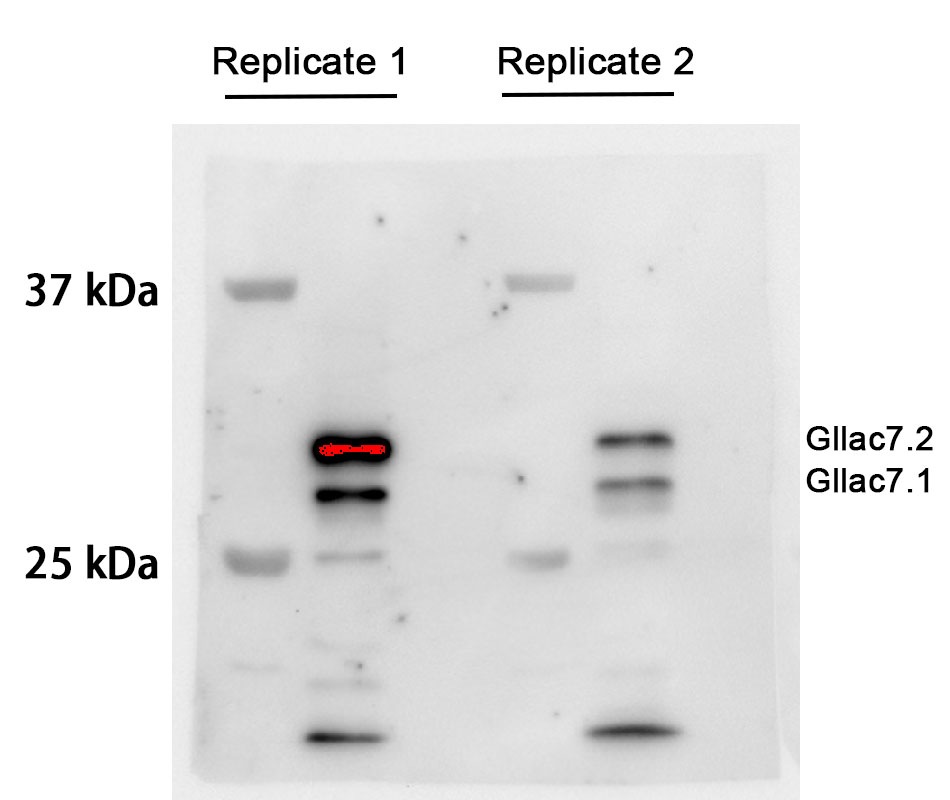

Supplement: Supplementary file 2 — Additional file 2: Fig. S6 Uncropped blots for Fig. 5E of anti-Gllac7. [file 12915_2024_2073_MOESM2_ESM.jpg]
